# Semantic Representation of Context for Description of Named Rivers in a Terminological Knowledge Base

**DOI:** 10.3389/fpsyg.2022.847024

**Published:** 2022-08-18

**Authors:** Juan Rojas-Garcia

**Affiliations:** Department of Translation and Interpreting, University of Granada, Granada, Spain

**Keywords:** named river, frame-based terminology, terminological knowledge base, analysis of predicate-argument structure, semantic network, thematic description, specialized knowledge representation, geographic contextualization

## Abstract

The description of named entities in terminological knowledge bases has never been addressed in any depth in terminology. Firm preconceptions, rooted in philosophy, about the only referential function of proper names have presumably led to disparage their inclusion in terminology resources, despite the relevance of named entities having been highlighted by prominent figures in the discipline of terminology. Scholars from different branches of linguistics depart from the conservative stance on proper names and have foregrounded the need for a novel approach, more linguistic than philosophical, to describing proper names. Therefore, this paper proposed a linguistic and terminological approach to the study of named entities when used in scientific discourse, with the purpose of representing them in EcoLexicon, an environmental knowledge base designed according to the premises of Frame-based Terminology. We focused more specifically on named rivers (or potamonyms) mentioned in a coastal engineering corpus. Inclusion of named entities in terminological knowledge bases requires analyzing the context that surrounds them in specialized texts because these contexts convey specialized knowledge about named entities. For the semantic representation of context, this paper thus analyzed the local syntactic and semantic contexts that surrounded potamonyms in coastal engineering texts and described the semantic annotation of the predicate-argument structure of sentences where a potamonym was mentioned. The semantic variables annotated were the following: (1) semantic category of the arguments; (2) semantic role of the arguments; (3) semantic relation between the arguments; and (4) lexical domain of the verbs. This method yielded valuable insight into the different semantic roles that named rivers played, the entities and processes that participated in the events educed by potamonyms through verbs, and how they all interacted. Furthermore, since arguments are specialized terms and verbs are relational constructs, the analysis of argument structure led to the construction of semantic networks that depicted specialized knowledge about named rivers. These conceptual networks were then used to craft the thematic description of potamonyms. Accordingly, the semantic network and the thematic description not only constituted the representation of a potamonym in EcoLexicon, but also allowed the geographic contextualization of specialized concepts in the terminological resource.

## Introduction

In linguistics, the convention has been established that a common noun, such as *river*, designates a category or class of individuals. Therefore, the meaning of *river* can be factorized to identify the multitude of individual rivers that are designated by this word. Nonetheless, a proper name, such as *Nile River*, is seen as the linguistic representation of an individual, namely, a unique entity in the world. Hence, *Nile River*, as a member of the class evoked by *river*, can be described but not defined. Based on such considerations, Sager (1990, p. 70) remarks that a proper name holds an individualizing value, whereas a common noun fulfills a classificatory function.

In the philosophy of language, a proper name is generally conceived, albeit with variations, as a linguistic expression that designates one and only one entity in the world because a fixed relation between the linguistic expression and that entity in the world can be established. From this basic conception, two schools of thought have emerged, namely, Referentialism and Predicativism. Referentialists defend that the only semantic function of a proper name is to designate an individual, and this referent constitutes its semantic content (Mill, 1843/2002, p. 21–22; Frege, 1892/1952, p. 54; Russell, 1905/1988; Wimmer, 1973/2011, p. 77; Kripke, 1980). In contrast, predicativists depart from this conservative stance, and start from the premise that a proper name is a type of common noun. They thus argue that the semantic function of a proper name is to designate properties of an individual, and that this set of properties comprises its semantic content (Quine, 1960; Burge, 1973; Elugardo, 2002; Matushansky, 2008; Fara, 2015).

The semantics of proper names thus remains a controversial issue, despite the fact that there is hardly any conceivable aspect which has not been exhaustively reconsidered. A comprehensive survey of the different philosophical and linguistic points of view can be found in Van Langendonck (2007, Ch. 1). However, in the following section “Proper Names in Linguistics,” we hold a brief discussion on the topic to emphasize its complexity and contextualize the real objective of this paper.

Our aim is thus to propose a linguistic and terminological approach to the study of named entities when used in scientific discourse, with the purpose of representing them in EcoLexicon (Faber et al., 2016; San Martín et al., 2020). This is a digital terminological knowledge base (TKB) on environmental sciences, designed according to Frame-based Terminology (Faber, 2012, 2015), the theoretical framework of this research. Frame-based Terminology is a cognitive theory of terminology that contextualizes concepts in frames, also called semantic networks or knowledge structures in this paper, and is based on corpus analysis. It is worth clarifying that a TKB is a resource that describes “the concepts and terms of specialized knowledge domains for users with linguistic and/or cognitive needs” (Faber and León-Araúz, 2016, p. 2), namely, it represents specialized knowledge either in a relational database or in an ontology (Temmerman and Kerremans, 2003; Roussey et al., 2018, p. 228), and contains specialized concepts with their definitions, the semantic relations that link them, and terms that lexicalize concepts in different languages or language communities (Condamines, 2018, p. 338).

This study focuses on potamonyms (i.e., the proper name of rivers, according to Room, 1996, p. 84–85), and analyzes the predicate-argument structure of sentences that mention named rivers in a coastal engineering corpus. It should also be pointed out that the methods to analyze potamonyms could be applied to named entities in other specialized domains, such as planets in astronomy, named bays and beaches in coastal engineering, named volcanos in vulcanology, named lakes and wetlands in limnology, named islands in nisology, and named rivers in potamology.

It is our assertion that named rivers, in the coastal engineering domain, have meaning—not only a referential function—, which is encyclopedic in nature according to cognitive linguistics (Evans, 2019, Ch. 15). This meaning thus encompasses dictionary knowledge (i.e., the lexical meaning of the term *river*) and encyclopedic knowledge, which corresponds to the specialized knowledge that coastal engineering texts convey about named rivers. In fact, named rivers hold a large number of semantic relations (e.g., *causes*, *improves*, *takes_place_in*, or *has_function*) that link them to other knowledge units, or terms, in the coastal engineering domain, as shall be seen. These terms correspond to a wide range of features, such as processes (e.g., *sediment supply*, *salinity intrusion*, *siltation*, and *freshwater input*), entities (e.g., *salt march*, *soft mud*, *dam*, and *jetty*), and attributes (e.g., *discharge rate*, *evaporation*, *sediment load*, and *runoff*). All of these designate concepts that are directly related to a named river. These concepts highlight and reinforce the specific nature and behavior of a named river, and differentiate it from other named rivers. Examples of this are provided in sections “Named Entities in Terminology” and “Results.”

In specialized discourse, verbs are means of such features to each named river. These verbs, which function as relational constructs, lead to the creation of a *semantic network*, or *semantic frame*, that represents specialized knowledge about a named river in the coastal engineering domain. This semantic network takes the form of a set of situational elements, namely, concepts and the semantic relations that link them.

For this reason, named rivers should have a *thematic description* in TKBs on the environment. The thematic description of a named entity is a textual explanation crafted from its semantic network, which depicts its relational behavior in a specialized domain. The thematic description pertains to the specialized domain in which the named entity has been analyzed because of its multidimensionality (Rogers, 2004), which involves that it is described depending on perspective and subject fields. Consequently, the same named river could have more than one description in a terminological resource, based on contextual constraints. One of the most important types of contextual constraint in terminology are the thematic constraints imposed by a knowledge domain, such as coastal engineering, hydrology, or potamology. As a result, the thematic description of a named entity is similar to the flexible approach to terminological definitions to represent thematic variation that proposed San Martín (2021). As an example of thematic description, that of the Salinas River (in California) in the coastal engineering domain, which is provided later, can be usefully summarized as follows: *Sea level rise is causing dune erosion of Monterey Bay beaches to progress at such a high rate that the sediments discharged by the Salinas River are not enough to alleviate the coastal erosion of the bay*.

It goes without saying that the thematic description of a proper name is not to be confused with the *definite description* of a proper name, to which grammarians, such as Quirk et al. (1985, p. 294), and philosophers of language, such as Russell (1905/1988), allude. The definite description of a proper name is a noun phrase, or paraphrase, that also makes reference to the same unique entity in the world to which the proper name refers. For instance, for the proper name *Nile River*, a definite description could be *the most important river to Ancient Egypt*; for the proper name *Joe Biden*, a definite description could be *the President of the United States in June 2022*; and the noun phrase *this odd neighbor* acts as a definite description when singles out an individual in a situated context.

Inclusion of named entities, such as named landforms, in TKBs requires analyzing the context that surrounds them in specialized texts because these contexts transmit specialized knowledge about named entities. Applying the proposal by Faber and León-Araúz (2016) for the parameterization of context, this paper thus analyzes the local syntactic and semantic contexts that surround potamonyms in coastal engineering texts, and describes the semantic annotation of the predicate-argument structure of sentences where a potamonym is mentioned. The semantic variables annotated are: (1) Semantic category of the arguments; (2) semantic role of the arguments; (3) semantic relation between the arguments; and (4) lexical domain of the verbs. The findings prove that this linguistic and terminological approach to the study of named entities in scientific discourse facilitates their representation in a TKB designed according to the framework of Frame-based Terminology.

More specifically, the results, on the one hand, allow us to draw conclusions on how each lexical domain of the verbs employed in the context of potamonyms is configured, namely, the specific combination of semantic roles and categories, and the semantic relation encoded by their different patterns of combination. This method also provides valuable information on the different semantic roles named rivers play in the coastal engineering domain, the entities and processes that participate in the events educed by potamonyms through verbs, and how they all interact.

On the other hand, since arguments are specialized terms and verbs are relational constructs, the analysis of argument structure leads to the construction of semantic networks that depict specialized knowledge about potamonyms in the coastal engineering domain. These conceptual networks are then used to craft the thematic description of potamonyms. Accordingly, the semantic network and the thematic description not only constitute the representation of a potamonym in EcoLexicon, but also allow the geographic contextualization of specialized concepts of the coastal engineering in the terminological resource.

The geographic contextualization of a specialized concept should provide a context representation, in the form of a semantic network, that covers a background situation in which the concept is embedded. In this sense, the geographic contextualization we are referring to consists in viewing the specialized concept from a situation in which the concept is related to specific named geographic entities, such as rivers and bays, because it is involved in an environmental problem which affects those geographic entities. For instance, the geographic contextualization of the sea level rise concept in the coastal engineering domain, as shall be seen in the section “Results,” would show a semantic network with situational elements (i.e., concepts and semantic relations) that would facilitate to represent and understand that sea level rise is causing dune erosion of Monterey Bay beaches to progress at such a high rate that the sediments discharged by the Salinas River are not enough to alleviate the coastal erosion of the bay.

The remainder of this paper is organized as follows. The section “Proper Names in Linguistics” summarizes different viewpoints in linguistics and the philosophy of language regarding the semantics of proper names. The section “Named Entities in Terminology” deals with the lack of named landforms in environmental terminology resources, gives reasons for this oversight, explains why named landforms should be included in terminological resources, and describes a set of principles to address the issues of categorization and inheritance, which arise upon representing named entities in a TKB. In section “Semantic Analysis From the Perspective of Frame-Based Terminology,” the fundamental premises of Frame-based Terminology are explained, according to which the analysis of local syntactic and semantic contexts of potamonyms in coastal engineering texts is performed. The section “Materials” describes the coastal engineering corpus where the potamonyms are mentioned, the Geonames database for the automatic recognition of potamonyms in the corpus, and the INCEpTION tool for the semantic annotation of local contexts. The section “Methodology” details the semantic variables annotated in the corpus, their values, and the inter-annotator agreement. The section “Results” reports the findings of the analysis of local contexts with regard to the semantic roles played by potamonyms, the semantic relations they hold with other arguments in the sentences, the semantic configuration of predicate lexical domains, the construction of two river-evoked semantic frames, the thematic description of the named rivers in both semantic networks, and the geographic contextualization of two specialized concepts integrated in those frames. Critical reflection on the semantic behavior of named rivers in the coastal engineering domain is provided in the section “Discussion.” Finally, the section “Conclusion” presents the conclusions derived from the semantic analysis of potamonyms, as well as plans for future research.

## Proper Names in Linguistics

Although lexicographers generally tend not to include proper names in conventional dictionaries, they do compile special dictionaries for them (e.g., *Placenames of the World* dictionary by Room, 2013). This indicates that most linguists believe proper names to be linguistic units that pertain to the lexicon of a language (Gardiner, 1940, p. 32–34; Quirk et al., 1985, p. 288; Cruse, 2000, p. 315–318; Levinson, 2003; Bennett and Agarwal, 2007; Tenbrink, 2007; Stock et al., 2019). Nevertheless, they are “semantically different from so-called appellative words (roughly corresponding to common nouns), so that we need different techniques and kinds of description for the meanings of proper names versus appellatives.” (Evans and Wimmer, 1990, p. 261). This need for a novel approach, which is more linguistic than philosophical, to the description of proper names is also recognized by other scholars.

The philosophers of language Strawson (1974/2004) and Searle (1969) came out in favor of a pragmatics and discourse view of proper names, and stressed that philosophers should consider the usage of proper names in natural language discourse, rather than only focusing on decontextualized short sentences or parts of sentences, for the elaboration of theories of proper names. Strawson’s and Searle’s claim possibly stemmed from the fact that context was systematically neglected in linguistic accounts for a long time since it was regarded as being too chaotic to be objectively described (Ervin-Tripp, 1996, p. 35).

It is thus hardly surprising that, as observed by the linguist Sjöblom (2006), the status of proper names in linguistics has not been satisfactorily addressed because the issue has been dominated by the philosophy of language. Hence, from the perspective of cognitive linguistics, she asserts that proper names are words that have meanings because they are inserted into the network of meanings that exists in our mind. In this way, Sjöblom’s (2006) view is in line with the principles of Frame-based Terminology (Faber, 2012, 2015), the theoretical framework of this paper.

Similarly, the onomastician Van Langendonck (2007, p. 2–3) states that “theoretical linguists have often treated proper names as the poor cousin of other grammatical categories. […] Onomasticians, however, have sometimes forgotten that proper names are part of the system of natural languages. Both onomasticians and linguists should be aware of the fact that proper names are words which deserve linguistic attention in the first place.”

## Named Entities in Terminology

### Theoretical Principles for the Representation of Named Entities in Frame-Based Terminology

As previously mentioned, for the representation of a named entity in a TKB, we propose both the construction of a semantic network that reflects the relational behavior of the named entity with other concepts in a specialized domain, and a thematic description that is a textual explanation of the relational behavior of the named entity, elaborated from its semantic network. This is supported not only in the cognitive linguistics approach adopted by Sjöblom (2006) for proper names, but also in the theory of proper names formulated by Searle (1983).

Searle (1983, Ch. 9) points out that the reference made by a proper name not only includes some kind of necessary and sufficient knowledge about the referent (e.g., its semantic category), but also descriptive knowledge on peripheral aspects related to the referent, provided that this conceptual content helps text senders to refer. Namely, everything that text senders semantically know about the referent helps them to accomplish their intention to refer. In this sense, Searle’s view is in consonance with Michalski (1991), who states that the context of a concept (i.e., a named entity in our case) is the set of concepts that contribute significantly to describe its intended features.

Thus, the semantic network of a named entity, on the one hand, must represent the conceptual structure that underlies its usage in specialized discourse, according to Sjöblom (2006); and on the other hand, the network must be endowed with the explanatory adequacy that Searle (1983, Ch. 9) postulates. These two principles for the construction of the semantic network of a named entity substantiate Frame-based Terminology (see section “Semantic Analysis From the Perspective of Frame-Based Terminology”). Therefore, a terminological resource designed according to this framework enables users to understand the relevance of a named entity for a subject field by giving them access to the necessary information to activate the knowledge structure in which the named entity is integrated. In this way, users can acquire background knowledge about the named entity necessary in communicative situations, such as specialized translation (Faber, 2012).

### Lack on Named Landforms in Environmental Terminology Resources

In terminology work, the description of named entities is a theoretically accepted activity, as evidenced by Sager (1990, p. 68–71), and the international standard for terminology work developed by the International Organization for Standardization (ISO) (2009, p. 36–37) (ISO 704: 2009). To ensure greater clarity, it is worth mentioning that this standard distinguishes between *proper name* and *appellation* for the designation of an *individual concept* (i.e., unique entity in the world, also referred to as individual entity in the standard, or named entity in this study). Accordingly, an appellation corresponds to a definite description (see section “Introduction”), used in a subject field, to designate an individual concept. For instance, the appellation (or definite description) *the United Nations Commissioner for Human Rights in June 2022* and the proper name *Michelle Bachelet* designate the same individual concept; the appellation *Il Duce* and the proper name *Benito Mussolini* also designate the same individual concept.

However, on a practical level, named landforms, such as rivers, bays, and beaches, are not represented in terminological resources on the environment. In our opinion, reasons for this absence include the following:

1. Firm preconceptions, rooted in philosophy, as to what named entities are, have presumably led to named landforms (e.g., Salinas River, Monterey Bay, Sunset Beach) being regarded as mere instances, with only a referential function, of categories such as river, bay, or beach. Their relational behavior with other concepts in a specialized knowledge domain has never been semantically described in any depth. Therefore, terminologists have been inclined to believe that the inclusion of the concepts of river, bay, or beach was sufficient. This belief doubtlessly applies to other named entities in specialized discourse as well.
2. Even though the inclusion of named landforms (and, in general, relevant named entities to a subject field) is justified, their semantic representation depends on knowing which concepts are semantically related to each of them, how those concepts are linked to each other, and which semantic relations should be included in the semantic network of each named landform. This is evidently a time-consuming task since terminologists rarely use natural language processing systems beyond corpus query tools such as Sketch Engine (Kilgarriff et al., 2004). On the other hand, although terminologists apply those natural language processing systems, the validation of the (semi-)automatically extracted information about a single named entity before storing it in a TKB is also a labor-intensive task.
3. The lack of clear guidelines for terminologists about how to deal with named entities has meant that their representation in TKBs is not a priority. In fact, although the relevance of named entities to certain specialized domains has been highlighted by prominent figures in the discipline of terminology (Sager, 1990, p. 68–71; Faber and León-Araúz, 2014; Faber, 2015, p. 26–27; L’Homme, 2020, p. 60–61), as far as we know, no research work has yet addressed in any depth how the description of a named entity, significant to a subject field, should be crafted in terminological resources.

Named landforms, among other named entities, are frequently found in specialized texts on environment. However, their representation in specialized knowledge resources has received little research attention. This is evident by the lack of named landforms in terminological resources for the environment such as EcoLexicon,^1^ DiCoEnviro,^2^ GEMET,^3^ or FAO Term Portal.^4^

In contrast, AGROVOC^5^ includes a list of named landforms with hyponymic information (only the semantic relation *type_of*), whereas ENVO^6^ provides descriptions of named landforms with only geographic details (e.g., geographic coordinates, and rivers that discharge into a certain bay), and minimal semantic information consisting of the relations *located_in*, and *tributary_of* in the case of named rivers and bays. Although the ENVO resource includes named landforms with their descriptions, these correspond to general knowledge focused on geographic data. This type of information does not permit users to understand either the pertinence of a named landform to a certain domain of specialized knowledge such as coastal engineering, hydrology, or sedimentology, or what relation the named landform holds to specialized concepts of a subject field.

### Why Named Landforms Should Be Included in Terminological Resources

So far, most TKBs have limited themselves to representing concepts such as river, bay, or beach, on the questionable assumption that the concepts linked to each of them are also related, respectively, to all named rivers, bays, and beaches in the real world. This issue is evident in the following explanation of forcing mechanisms acting on suspended sediment concentration (SSC) in rivers and bays.

According to Moskalski and Torres (2012), temporal variations in the SSC of bays and rivers are the result of a variety of forcing mechanisms. River discharge is a primary controlling factor, as well as tides, meteorological forcing (i.e., wind-wave resuspension, offshore winds, storm, and precipitation), and human activities. Various of these mechanisms tend to act simultaneously. However, the specific mix of active mechanisms is different in each bay and river. For example, SSC in the *San Francisco Bay* is controlled by spring-neap tidal variability, winds, freshwater runoff, and longitudinal salinity differences; whereas precipitation and river discharge are the mechanisms in the *Suisun Bay*. In the *Yangtze River*, SSC is controlled by tides and wind forcing; whereas river discharge, tides, circulation, and stratification are the active forcing mechanisms in the *York River*.

Consequently, in a specialized knowledge resource, a comprehensive list of forcing mechanism concepts semantically linked to the river and bay concepts, would not accurately represent the knowledge really transmitted in specialized texts because such a representation would inappropriately establish that all forcing mechanisms acting on SSC occur in all the rivers and bays in the world.

Indeed, as shall be seen in the section “Results,” each named river in the coastal engineering domain educes a semantic network depicting a specific topic, associated with an environmental problem. Moreover, each of these river-evoked frames shows a different set of situational elements (i.e., concepts and semantic relations), a fact that proves the specific relational behavior of each named river. Therefore, given that each named river exhibits a specific relational behavior in specialized discourse, it is our assertion that TKBs should include the representation of named landforms and whatever named entity deemed to be relevant to a subject field.

### Categorization and Inheritance

Upon representing named entities in a TKB, categorization and inheritance issues arise, which require basic principles to be laid down, similar to those applied to the representation of named rivers in EcoLexicon. These principles are based on research into the human categorization of spatial and non-spatial entities by Barsalou (1985), Davies (2009, 2020), and Rosch et al. (1976).

Rosch et al. (1976) provided evidence that categorization does not lead to clearly delimited categories of elements with shared properties based on necessary and sufficient conditions, but rather to categories with a graded structure and fuzzy boundaries, in which some members are deemed more prototypical than others.

In turn, Barsalou (1985) established that, in addition to the resemblance to a prototype, there exist other three factors that determine the graded structure of categories: (1) goal-based ideals (i.e., features related to function or purpose of a concept in a context); (2) frequency of instantiation (i.e., how often a person has understood a concept to pertain to a category); and (3) personal familiarity with a concept. He found that the prototypicality of a concept depends on context and type of category, namely, taxonomic category (e.g., types of estuary, and means of transport), or goal-derived category (e.g., aesthetics of a place as well as its function). As such, prototypicality exerts more influence on taxonomic categories, whereas goal-based ideals and frequency of instantiation act on both types of category.

Goal-based ideals have been found to play a major role in determining the graded structure of even natural-kind categories despite being taxonomic, for instance, in the categorization of trees (Lynch et al., 2000) and birds (Burnett et al., 2005). Furthermore, in both research studies, knowledge domain experts employed goal-based ideals in contextualized categorization tasks (related to a utilitarian view and purposes rather than proximity to prototypes) much more frequently than novice students. At the highest level of expertise, personal familiarity had a greater impact than prototypicality. Similarly, in an fMRI study of expert-novice differences in the identification of geological field instruments, Faber et al. (2014) found that, unlike novices, experts activated, among other brain regions, those involved in the representation of context and the codification of meaningful contextual associations.

The environmental psychologist, Clare Davies, upon replicating experiments on human categorization by Barsalou (1985) for the case of geographic entities, found that named places are regarded as concepts (Davies, 2009) and may be treated cognitively as semantic categories of locations (Davies, 2020, p. 9–10), a fact that has been long considered intuitively reasonable in the discipline of Geography (Montello, 2003). In other words, in human mind, a place behaves as a semantic category, and each feature linked to the place is thus stored as a semantically related exemplar of it. Even physical items located within the place are cognitively processed as exemplars of the place, not just as contiguous points in space. In Davies’s (2020) work, named places showed several of the same characteristics of categorization as the aforementioned studies of non-spatial objects. Namely, places are fuzzy categories, and influenced by context, expertise, goal-based ideals, prototypicality, and physical or spatial similarity.

Although Davies (2020) did not explore in her experiments aspects such as hierarchical structure and the presence of a basic level in place categories (Rosch, 1978; Murphy and Lassaline, 1997), she emphasized that work in Geographic Information Science has provided evidence of the presence of both characteristics (Hirtle and Jonides, 1985; Lloyd et al., 1996; Edwardes and Purves, 2007).

Regarding the categorization and inheritance issues that arise when including named rivers in EcoLexicon, the following principles are adopted, based on the previously discussed research.

For purposes of specialized knowledge representation, a named entity, such as a named river (e.g., the *Salinas River*, in California), should be considered to be a subordinate concept of the river concept in virtue of its specific relational behavior with specialized concepts within a subject field. Accordingly, the named river inherits from the river concept the properties that allow it to be identified as a member of the river category within a cultural community. Therefore, other named rivers, such as the *Dee River* (in the United Kingdom), would be considered a cohyponym of the *Salinas River*.

Consequently, if each named river is related to a distinct set of specialized concepts within a subject field, this poses the challenging question as to what specialized concepts are then to be linked to the river concept, as superordinate concept, within the same subject field in a knowledge resource. Although there is no simple answer since the problem can be approached from diverse points of view and disciplines, we offer three possible solutions.

Firstly, one could opt not to link any specialized concept to the river concept, only to named rivers. As such, named rivers would only inherit from river the properties that allow them to be identified as members of the river category.

The second solution is based on Rosch et al. (1976), and Barsalou (1985). Namely, those specialized concepts (also referred to as features), which are related to the majority of named rivers in our sample, might also be linked to the river concept. Hence, the named rivers, as subordinate concepts, would also inherit from river the features that are common to the majority. The drawback is that some named rivers would inherit features that are not related to them. In this situation, the factors that determine the graded structure of the river category become important. For this reason, named rivers could be regarded as more or less prototypical members of the river category according to Rosch et al. (1976); or alternatively, their categorization would be based on the goal-based ideals of Barsalou (1985). In this way, these named rivers would be prevented from inheriting those features that are not related to them. Thus, the TKB should implement a mechanism to impose inheritance restrictions.

The third solution consists in linking to the river concept all features related to the named rivers in our sample. However, the links would be numerically weighted according to the commonness of the features among the named rivers. In other words, the more named rivers associated with a feature, the greater the link weight for that feature. In this way, the river concept would reflect all the potential features that could be activated depending on context. This is a solution in consonance with the major role of context in the selective activation of previously stored knowledge (Croft and Cruse, 2004, p. 75). However, this could also produce an excessive information load for users of a terminological resource, since *river* can rarely activate all those features at the same time in a specific context. For this reason, users could set a threshold for the weights of the links, so that only the features whose link weight is greater than the threshold would be shown in the semantic network of the river concept. This could be interpreted as a graded adhesion of the features to the river concept.

In the three solutions proposed, the features linked to named rivers are those that corpus data ascribe to each named river. Obviously, if corpus data do not associate a feature with a named river, this does not mean that the feature is not actually related to it. However, as the terminological resource becomes populated with data from an increasingly large corpus, this issue will become less critical. In any case, whatever the implemented solution, the final representation should be validated by experts in the field in which the named entities are analyzed.

### Cross-Cultural Conceptualizations of Landforms

Further explanations should also be provided with respect to cross-cultural differences in the conceptualization of landforms. The semantic content of words for parts of the physical world is determined by the cultures of the speakers (Sharifian, 2011). Consequently, landform terms, such as *river*, *mountain*, *bay*, and *wetland*, do not possess identical meanings in all languages (Smith and Mark, 2003). Culture and utility (i.e., affordance, understood as the resource that the environment offers people) also influence the categorization of landforms, not only intellectual interest (Smith and Mark, 2001). For instance, in the case of river, Bromhead (2018, Ch. 2) compares four languages and uncovers differences and common factors in the configuration of the river concept.

This paper focuses on the usage of named rivers in a coastal engineering corpus in English language. Therefore, the analysis of cross-cultural differences in conceptualization of this landform, in the same subject field, in other languages is outside the scope of this study. Notwithstanding, in future work, with the purpose of making EcoLexicon an inclusive resource sensitive to cultural variation, it will thus integrate different cultural views on specialized concepts of the environment. The cultural parameters with which this project will begin are the following: geographical origin, variations from each environmental discipline, and degree of specialization. As such, the cultural adaptation of the conceptual module of EcoLexicon will allow to contextualize the semantic networks of named rivers according to the cultural parameter of geographical origin.

## Semantic Analysis From the Perspective of Frame-Based Terminology

Frame-based Terminology (Faber, 2012, 2015), the approach applied in EcoLexicon and in this study, organizes knowledge in semantic frames, thereby creating non-language-specific representations. Such configurations are the conceptual meanings underlying specialized texts in different languages. This specification facilitates specialized knowledge acquisition because it relates entities and processes associated with a particular situation that is part of human experience (Barsalou, 2003). According to Frame Semantics (Fillmore, 2006), in order to understand the meanings of words in a language, it is first necessary to have knowledge of the semantic frames, or conceptual structures, that underlie their usage.

Frames have the advantage of making explicit both the semantic and syntactic behavior of specialized language units. This necessarily includes a description of semantic relations as well as a term’s combinatorial potential (Faber, 2009, p. 123). Frames conceptualize reality by means of a closed set of hierarchical relations, such as the hyponymic and meronymic relations *type_of*, and *part_of*; and non-hierarchical or associative relations, such as *causes*, *improves*, *results_of*, and *has_function*, which are domain-specific relations that make knowledge representation more meaningful and connected to reality since they show both multidimensionality and dynamism (Faber et al., 2009, p. 16; León-Araúz, 2009, p. 149, 176, and 184).

In summary, frames facilitate specialized knowledge acquisition and make knowledge representation more meaningful. These properties comprise what is called *explanatory adequacy* of a semantic network in Frame-based Terminology.

On the other hand, since a frame is activated by a linguistic item and the units in its cotext, its construction implies the semantic analysis of predicate-argument structures, which refer to the lexical representation of argument-taking lexical items (Levin, 2013/18). These are typically verbs and their nominalizations. The specification of the argument structure involves identifying the number of arguments that a lexical item can take, their syntactic expression, and their semantic relation, or semantic role, to the predicate.

Although syntactic expression is language-specific, semantic relations to the predicate are not. For that reason, what is important is not the syntactic realization of the predicate and its nominalization, but rather the combination of semantic roles and categories. In this way, the frame is generated by this combination of semantic roles and categories, and the relation between them (Faber and Cabezas-García, 2019, p. 202–204).

Consequently, this paper focused on the semantic analysis and annotation of sentences that mention potamonyms in coastal engineering texts. This permitted the subsequent construction of semantic frames that reflected the usage of named rivers in that domain for the purpose of representing them in EcoLexicon. These frames can function as interlingual representations, thereby facilitating their processing by computers (Boas, 2005; Segev and Gal, 2008; Baker, 2009; Pimentel, 2015), for instance, in machine-translation applications (Buendía-Castro and Faber, 2016), and computer-aided specialized translation (León-Araúz et al., 2020).

## Materials

### Corpus Data

The sentences that cite named rivers were extracted from a subcorpus of English texts on coastal engineering, comprising roughly 7 million tokens. This subcorpus was composed of specialized texts (scientific articles, technical reports, and Ph.D. dissertations), which amounted to 73.17% of the corpus size; and semi-specialized texts (textbooks and encyclopedias on coastal engineering), which constituted 26.83% of the corpus size. The total number of texts of the subcorpus was 2,249, whose publication data ranged from 1996 to 2018.

This subcorpus in costal engineering is part of the English EcoLexicon Corpus,^7^ which currently contains over 100 million tokens in English and is focused on the environmental domain. It was manually compiled for the development of the EcoLexicon database. We refer the reader to León-Araúz et al. (2018) for a detailed description of its design and compilation criteria.

The coastal engineering domain was chosen for the semantic representation of named rivers because it is an interdisciplinary science that studies coastal processes, both natural and human-induced, for the design of maritime works and environmental recovery projects. Since one of the functions of coastal engineering is shore protection against erosion and flooding, coastal engineers design coastal defense structures such as breakwaters, dikes, and revetments. They also may envisage non-aggressive solutions such as dune restoration, artificial nourishment, and revegetation.

According to the Coastal Sediment Management Workgroup (2009, p. 1), although beach erosion is a natural process, human activities have reduced the natural supply of sediments to the coast and have thus modified alongshore sediment transport. For instance, dams block the transport of sediment through rivers, thus decreasing the downstream transport of sediments that reach bays; coastal structures, such as groins and breakwaters, alter the transport of sediment along the coast; harbors in bays trap sediment and also modify its transport patterns along the coast to naturally nourish beaches. Consequently, since the nature of coastal and environmental problems vary widely depending on the location, and the proper solution needs specific evaluation (Coastal Engineering Research Center, 1984, p. 1), the semantic analysis of individual named rivers (among other geographic entities such as named beaches and bays) is required for their accurate representation in TKBs on the environment.

### GeoNames Geographic Database

Automatic detection of the named rivers mentioned in the corpus was performed with a dataset obtained from the GeoNames geographic database (Ahlers, 2013). GeoNames^8^ has over 10 million proper names for 645 different geographic categories, such as bays, beaches, rivers, deltas, estuaries, river basins, river valleys, mountains, bridges, and populated places. For each entity, information is stored regarding its normalized designations, alternate designations (including other languages than English), latitude, longitude, and location name. A daily GeoNames database dump is publicly available for download in the form of a large worldwide text file, which was used for the recognition of named rivers in the corpus.

### INCEpTION Annotation Tool

The INCEpTION tool (Klie et al., 2018) is a state-of-the-art annotation platform for semantic annotation (e.g., semantic frame annotation, knowledge base population, and entity linking, *inter alia*), which integrates machine learning capabilities, knowledge management, an intuitive user interface, and the ability to manage multiple annotation projects with several users involved.

To improve the manual annotation process, INCEpTION system makes use of predictive machine learning algorithms, which continuously monitor the labels attached by the user to provide annotation suggestions that the user can accept or reject. In this way, the feedback provided, and the changes made during the manual annotation process flow to the algorithm, which retrains the predictive model to update the annotation suggestions.

For knowledge management, INCEpTION allows users to create and edit an internal RDF-based knowledge base by annotating facts (i.e., triplets formed by a subject, a predicate, and an object) in the predicate-argument structure of sentences in corpus texts. In doing so, a domain-specific knowledge base can be constructed and expanded as part of the annotation task. The population of this internal knowledge base can then be used for fact-linking. In addition, external knowledge bases, such as Wikidata, DBPedia, ENVO, or EcoLexicon, can be accessed *via* SPARQL. These external resources enable users to perform knowledge-driven annotations such as entity linking, which signifies linking terms, mentioned in texts, to the corresponding concepts, stored in a knowledge base, which are designated by those terms.

The INCEpTION annotation scheme organizes annotations into layers, which represent the features to be annotated in a project (e.g., semantic roles, semantic categories, or named entity types) and their labels (e.g., the labels agent, patient, and theme for the semantic role feature). Any number of layers can be defined, which can be spans or relations between spans. Each layer can also have any number of features, which can be strings, numbers, Booleans, concept references, or references to other annotations.

**Figure 1:**
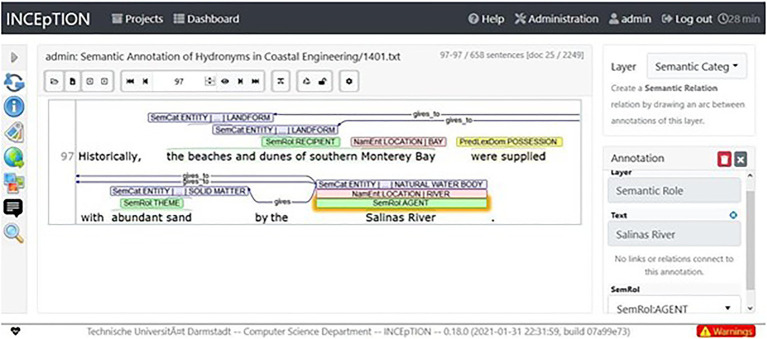
Annotation user interface of the INCEpTION tool, where the semantic annotation of the predicate-argument structure of sentences mentioning a named river in the Coastal Engineering corpus was carried out. In the example sentence, the *Salinas River* and the *Monterey Bay* are mentioned. Image reproduced with the permission of Dr. Richard Eckart de Castilho, INCEpTION project lead at the The Ubiquitous Knowledge Processing (UKP) Lab at the Department of Computer Science, Technische Universität Darmstadt.

Figure 1 shows the annotation user interface of the INCEpTION tool and the semantic annotation of the predicate-argument structure of a sentence, mentioning the *Salinas River*, in the coastal engineering corpus. The annotation scheme designed for our semantic annotation task is explained in the following section.

## Methodology

### Recognition of Named Rivers

In the experiments by Stokes et al. (2008) which compared different natural language processing methods to detect toponyms in texts, it was found that a simple approach employing a gazetteer (i.e., a dictionary of proper names for geographic entities) to recognize the presence of toponyms outperformed other sophisticated methods. Hence, we applied the matching of named entities, using the GeoNames geographic database to identify the named rivers, deltas, estuaries, river basins, river valleys, and river mouths mentioned in the corpus.

The corpus texts were tokenized, tagged with parts of speech, lemmatized, and lowercased with the Stanford *CoreNLP* package (Manning et al., 2014) for the R programing language (R Core Team, 2021). Then, both normalized and alternate names of rivers, deltas, estuaries, basins,^9^ valleys, and mouths in the GeoNames database dump were searched in the lemmatized corpus. A total of 783 different designations were recognized and listed.

Most designations cited in the corpus were in GeoNames (97%), while others were identified by manual inspection (3%). Namely, with a view to estimating the capability of GeoNames for recognizing named landforms in the corpus, we first queried the corpus documents that contained the terms *river*, *delta*, *estuary*, *basin* (and the synonyms *catchment* and *watershed*), *valley*, and *mouth*. Then, we listed all the potamonyms that were manually identified in those corpus documents. In this way, we could ascertain that GeoNames matched 97% of the potamonyms in our list. This high performance of GeoNames allows us to trust that it will also be able to match a substantial percentage of other named landforms, relevant to coastal engineering, that are mentioned in the corpus, such as named bays, beaches, and coasts, which will be analyzed in future work.

Anaphoric elements that referred to a river, delta, estuary, basin, valley, or mouth were replaced by the corresponding full designations in the lemmatized corpus. For this task, the automatic anaphora resolution function of the *CoreNLP* package was used.

Since various designations can refer to the same river because of syntactic variation (e.g., *Nile River* and *River Nile*), and orthographic variation (e.g., *Yangtze* and *Yangtse River*), the variants were identified to give them a single designation in the corpus. Once the variants were normalized in the lemmatized corpus and joined with underscores, the number of named rivers, deltas, estuaries, basins, valleys, and mouths was 676.

The mouths of the 360 rivers mentioned in the corpus were shown on a map with color-coded rectangles that represented their frequency in the corpus. Their latitudes and longitudes were retrieved from the GeoNames database dump. This reflected the representativeness of the corpus in reference to river locations and their number of mentions. The named rivers were in a large number of countries, but the most cited rivers were located in the United States.

A critical issue was to disambiguate to which river with the same name the text referred to. Namely, although latitudes and longitudes could be retrieved from the GeoNames database dump, the same designation occasionally referred to rivers in different countries. For instance, the corpus only located the *Yellow River* in China. However, GeoNames indicated that rivers with the same name also existed in the United States, Canada, Ireland, and Papua New Guinea. Such cases had to be resolved by corpus queries.

**Figure 2:**
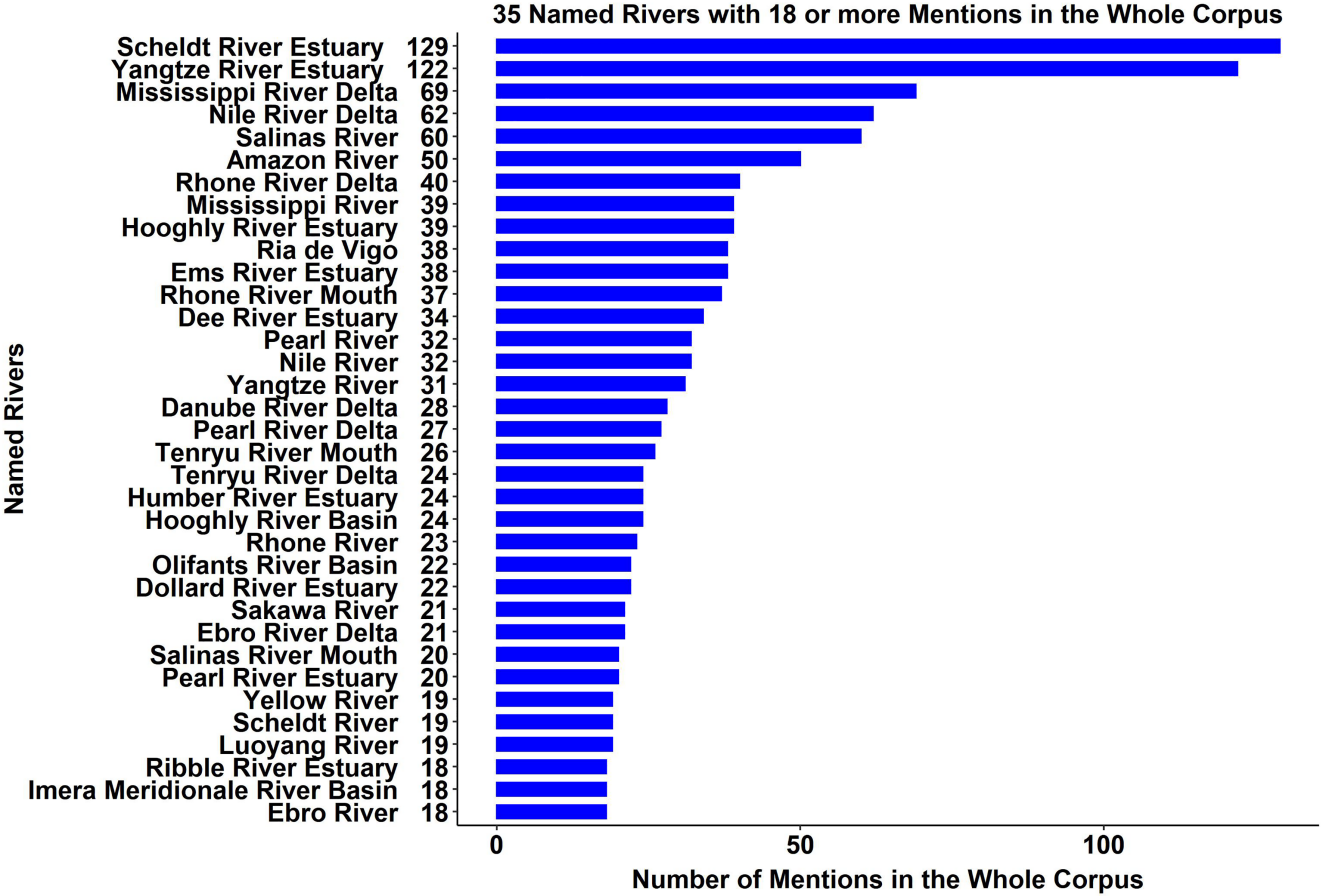
A sample of 35 designations of rivers and their number of mentions.

The occurrence frequency of the named rivers, deltas, estuaries, basins, valleys, and mouths ranged from 129 (*Scheldt River Estuary*) to only one mention (349 out of 676 designations). Figure 2 shows a sample with the 35 most frequently cited designations, along with their number of mentions.

### Semantic Annotation of Predicate-Argument Structures for Named Rivers

The 676 designations encompassed a total of 2,840 mentions of named rivers, deltas, estuaries, basins, valleys, and mouths in the corpus. We decided to analyze more than 50% of the mentions, which meant including in the sample the designations that appeared 8 or more times. This led to the semantic annotation of 1,694 mentions, which embraced rivers, deltas, estuaries, basins, valleys, and mouths. Accordingly, the sample of 1,694 annotated mentions represented 59.65% of the total number of mentions in the corpus.

For simplicity, named rivers, deltas, estuaries, basins, valleys, and mouths are henceforth referred to as named rivers or potamonyms. As such, the verbal and nominal predicates that occurred with named rivers in our corpus were first classified into the lexical domains defined by Faber and Mairal (1999). Then, the argument structure of the predicates was analyzed and annotated.

The set of 1,694 sentences from the corpus was annotated by three terminologists from the LexiCon research group at the University of Granada (Spain). They performed the semantic annotation of the predicate-argument structure of a sentence by assigning: (1) a lexical domain to the predicate; (2) a semantic role to the arguments of the predicate; (3) a semantic category to the arguments of the predicate; and (4) a semantic relation to the link between the potamonym and the other arguments in the sentence. In the following, these annotation categories are described, and examples of annotated sentences are provided later, in the section “Results.”

#### Predicate Classification in Lexical Domains

According to Faber and Cabezas-García (2019, p. 205), the most frequent verbs in our corpus, as part of the English EcoLexicon Corpus, are general language verbs (e.g., *accumulate*, *pollute*, *increase*, *discharge*, *supply*, and *drain*), which are also used in specialized texts.^10^ However, when they have terms for their arguments, this makes them domain-specific (L’Homme, 2003; Buendía-Castro, 2013). Even though verbs (especially general language verbs) have hardly been regarded as important in Terminology, they reflect how environmental entities interact (Buendía-Castro, 2013). In this sense, such verbs are also susceptible to classification into the lexical domains proposed by Faber and Mairal (1999), within the Functional-Lexematic Model (Martin, 1984/2017).

These authors propose, after analyzing over 10,000 verbs in the English language, a model for their lexical classification into domains based on the distinction between paradigmatic and syntagmatic relations. A lexical domain is thus formed by a hierarchy of verbs, all of which share the same nuclear meaning and syntax. The most prototypical verbs, or superordinate verbs, are those that have the largest combinatory potential from a semantic point of view.

In Frame-based Terminology, such verbs and their nominalizations provide the frames that characterize the actions and processes in the specialized field and link the semantic categories of the typical participants. In this regard, the majority of verbs in the English EcoLexicon Corpus were found to belong to the lexical domains shown in Table 1 (Faber and Cabezas-García, 2019, p. 206), used to annotate the predicates of our set of sentences.

**Table 1:** The most frequent lexical domains of environmental verbs (Faber and Cabezas-García, 2019, p. 206).

Lexical domain	Prototypical verb	Verb examples from the corpus
change	[to become/change]	*change, become, decrease, increase improve, transform, worsen, consolidate, influence, vary*
movement	[to move]	*carry, transport, transfer, introduce, go into, discharge, enter, drain, overflow*
existence	[to be/exist]	*prevent, produce, originate, occur, arise, develop, form, grow, initiate, result in*
possession	[to have]	*absorb, drain, distribute, lack, obtain, offer, provide, receive, supply, discharge*
position	[to be in a state/place/position]	*arrange, bound, confine, insert, place, remain, stay, deposit, accumulate, locate*
manipulation	[to use]	*manipulate, influence, control, treat, deal, manage, monitor*, *recycle, use*, *utilize*
action	[to do/make]	*perform, dam, respond, behave, make, operate, produce, work, construct, be under construction*
cognition	[to know/think]	*analyze, ascertain, assess, categorize, classify, compare, determine, estimate, underestimate, overestimate*
impact	[to hit/break]	*thresh*, *strike*, *slam*, *shatter*, *rupture*.

#### Semantic Roles

Specialized knowledge representation includes semantic properties that help to describe the nature of entities and processes. These semantic properties are reflected as the relations between a predicate and its arguments, which are typical semantic roles.

Although most linguists tend to believe that semantic roles exist, there is considerable disagreement as to their number, nature, and function (Ureña et al., 2013, p. 180). The set of semantic roles in this study largely coincided with those specified by Kroeger (2005, p. 54–55), and Thompson et al. (2009). Table 2 shows the list of the semantic roles used to annotate the arguments in our set of sentences.

**Table 2:** List of semantic roles and their definitions, based on Kroeger (2005, p. 54–55) and Thompson et al. (2009).

Semantic roles	Definition
agent	Entity/process that causes an action, whether intentionally or unintentionally.
result	Entity/process that has come about as a consequence of a voluntary/involuntary action.
patient	Entity which is acted upon, affected, or created; or of which a state, or change of state, is predicated.
theme	Entity which undergoes either a change of location or a change of possession. Also, entity whose location is being specified.
location	Spatial reference point of a process or an entity (the source, goal, and path roles are often considered to be subtypes of location).
recipient	Entity which receives or acquires something.
instrument	Entity used by an agent to perform some action. Instrument refers to the tools, machinery, and devices that are used to carry out human process events in the environment. However, natural entities can also use natural instruments.
time	Phrase that situates an event in time or with respect to another event.
rate	Phrase that describes changes in rate or level that occur as part of an event. In most cases, this role applies to the theme of an event.
manner	Phrase that describes the method or way in which a particular event is carried out.
description	Phrase that describes characteristics or behavior of the agent or theme of the event.
condition	Phrase describing the environmental conditions which must hold in order for the event to take place.
purpose	Process that specifies why another process occurs, i.e., specifications of some sort of aim, purpose, goal, or reason for the process occurring.

#### Semantic Categories

The concepts in EcoLexicon were classified into 153 semantic categories, hierarchically organized, and distributed in five categorization levels (Gil-Berrozpe et al., 2019). The most general level is composed of the three basic ontological categories, namely process (i.e., events extending over time and involving different participants), entity (i.e., physical and mental objects), and attribute (i.e., properties of entities and processes).

However, depending on the ontological nature of concepts, they can be subclassified in up to five levels of specificity. For instance, the semantic category of the beach size sand concept is mineral, placed on the fifth level of the category hierarchy entity > matter > solid matter > material > mineral.

From an ontological point of view, 15 categories were associated with attributes, 94 with entities, and 44 with processes. Accordingly, this hierarchically organized list of 153 semantic categories was used to annotate the arguments in our set of sentences. For a full list of the semantic category hierarchy and some examples of each category, see Gil-Berrozpe et al. (2019).

#### Semantic Relations

Conceptual description in EcoLexicon is based on the semantic category of concepts and their relational behavior. A fixed set of semantic relations, both hierarchical and non-hierarchical, was systematically defined by Faber et al. (2009) to make EcoLexicon a consistent resource at its different representational levels. These relations, with additional non-hierarchical relations specific to named rivers, are shown in Table 3, along with examples in the form of conceptual propositions (i.e., triplets consisting of two concepts and the semantic relation that links them). They were all used to annotate the semantic relation between the arguments in our set of sentences, specifically, the link of a named river to another argument in the same sentence. The non-hierarchical relations specific to named rivers are explained in the section “Results,” since they emerged from the semantic analysis of the predicate-argument structures.

**Table 3:** Semantic relations used during the annotation process.

**Relation category**	**Relation**	**Example**
Generic-specific	*type_of*	wind erosion *type_of* erosion
Part-whole	*part_of*	right bank *part_of* river
	*made_of*	groin *made_of* wood
	*delimited_by*	mesosphere *delimited_by* stratosphere
	*located_at*	soft mud *located_at* Mississippi River Mouth
	*takes_place_in*	consolidation of the land *takes_place_in* Mississippi River Mouth
	*phase_of*	pumping *phase_of* dredging
Non-hierarchical relations in EcoLexicon	*affects*	erosion *affects* coastal dune
	*causes*	Sacramento River *causes* freshwater inflow
	*result_of*	Tenryu River Delta *result_of* sediment supply
	*attribute_of*	discharge rate *attribute_of* Weser River
	*has_function*	Salinas River *has_function* sand supply
	*studies*	potamology *studies* surface current
	*measures*	optical backscatter sensor *measures* suspended sediment concentration
	*effected_by*	dredging *effected_by* dredger
Additional non-hierarchical relations for the semantic frames evoked by Named Rivers	*improves*	Salinas River Estuary *improves* water quality
	*worsens*	sea level rise *worsens* Salinas River Estuary
	*creates*	Mississippi River *creates* natural levee
	*becomes*	Saint Bernard River Delta *becomes* Chandeleurs Islands
	*gives*	Salinas River *gives* beach-size sand
	*gives_to*	Yangtze River *gives_to* Yellow Sea
	*receives*	Dee River *receives* sediment
	*receives_from*	Po River *receives_from* Po Plain
	*drains*	Santa Clara River *drains* watershed
*has_path*	*moves_over*	Salinas River *has_path*/*moves_over* riverbank
	*moves_into*	bed sediment load *has_path*/*moves_into* Yangtze River Estuary
	*moves_across*	Murray River *has_path*/*moves_across* Tertiary formation
	*transfers*	Weser River *transfers* sediment load
	*discharges_into*	Salinas River *discharges_into* Monterey Bay
	*places*	Mississippi River *places* soft mud
	*controls*	Ventura River *controls* sediment supply
	*applied_to*	NOAH model *applied_to* Mississippi River Basin

#### Annotation Scheme in the INCEpTION Tool

The INCEpTION annotation scheme organizes annotations into layers, which represent the features to be annotated in a project and their labels. We set five layers for 10 annotation features: (1) the first layer for the *predicate lexical domain* feature; (2) the second layer for the *semantic role*; (3) the third layer for the *semantic relation*; (4) the fourth layer to describe the named entity, which included two features, namely the *named entity* feature (initially, with the three basic labels location, organization, and person), and the *hydronym* feature to annotate whether the location was a bay, beach, coast, river, delta, estuary, river basin, river valley, or river mouth; and (5) the fifth layer for five features that stored, respectively, the five levels of the semantic category hierarchy.

#### Inter-Annotator Agreement

As previously stated, the annotation of the predicate-argument structures in the coastal engineering corpus was carried out by three terminologists. As for the inter-annotation agreement (Brezina, 2018, p. 87–92), *Cohen’s kappa* coefficient (*κ*; Cohen, 1960) showed a very good agreement for all annotator pairs (90% < *κ* < 98%, values of *p* < 0.05) in the initial annotations of semantic roles, categories, and relations, according to Krippendorff’s (2012) recommendations for text content analysis. A review of the differences between annotators showed no systematic pattern of disagreement. Given the nature of the judgment variables, the level of agreement was deemed acceptable. Notwithstanding, the disagreements in the original annotations were resolved based on discussion between the annotators to reach a consensus on the definitive annotations of semantic roles, categories, and relations.

For the initial annotation of predicates with lexical domains, the inter-annotation agreement was lower for all the annotator pairs (84% < *κ* < 88%, values of *p* < 0.05), indicating that this variable lent itself to alternative, though plausible, interpretations. A review of the differences between annotators showed that the lexical domains of movement and possession were more prone to confusion. The issues fundamentally arose from verbs that could potentially belong to more than one lexical domain, as Faber and Mairal (1999) already proved. To arrive at a consensus on the definitive annotations of lexical domains, the factorization of meaning from the Functional-Lexematic Model framework was applied to verbs, such as *drain* and *discharge*. The meaning factorization of *drain* is described in the following, as an example of the process used when there was disagreement between the annotators.

Although *drain* is a general language verb, it becomes a specialized verb in domain-specific texts when its arguments are filled with specialized knowledge units. As shall be seen, in some cases, the semantic content of its domain-specific arguments interacts with its base meaning to create a new sense that is appropriate for certain coastal engineering contexts (L’Homme, 2003; Faber and León-Araúz, 2016).

In the *Merriam-Webster Dictionary*, the intransitive use of *drain* can have four senses. However, only sense 2, *to discharge surface or surplus water*, was used in our sample of specialized contexts. Accordingly, *drain* belongs to the lexical domain of movement, as shown in sentence (1) of the sample:

1. The [Salinas River]_SemRol: **theme**_ *drains* into the [Monterey Bay]_SemRol: **location**|SemCat: **landform**_

Similarly, the transitive use of *drain* can have nine senses. Of these senses, only 2b, *to carry away the surface water of*, was used in our sample of specialized contexts. This means that transitive *drain* also belongs to the lexical domain of movement, as shown in sentence (2) of the sample:

2. Natural sediment supply within this region is defined by the [Ventura River]_SemRol: **agent**_ that *drains* large [watersheds]_SemRol: **patient|**SemCat: **landform**_

Therefore, sentences (1) and (2) maintain the base meaning of *drain* in general language. More specifically, they foreground the movement of water from one place to another, and thus convey the semantic role patterns theme + location, and agent + patient, respectively, where the semantic category of the location and patient arguments is landform.

Nonetheless, sentences (3) and (4) show how the semantic content of the domain-specific arguments of *drain* interacts with its base meaning to create a new sense, which is not used in the general language. In other words, sentences (3) and (4) do not highlight the movement of water, but rather foreground the change of possession of sediments or water from one entity (e.g., Po plain) to another (i.e., named rivers). Consequently, in both sentences, the verb *drain* belongs to the lexical domain of possession because its argument structure reflects the semantic role pattern agent + theme + recipient,^11^ where the semantic category of the theme argument is matter, and that of the recipient argument is landform, specifically, river.

3. Normally, eutrophic conditions are caused by [waters]_SemRol: **theme|** SemCat: **matter**_ *drained* by the [Po River]_SemRol: **recipient|** SemCat: **landform > river**_ from the highly inhabited and cultivated [Po plain]_SemRol: **agent|**SemCat: **landform**_
4. Not all the [sediments]_SemRol: **theme|** SemCat: **matter**_ *drained* by the [Dee River]_SemRol: **recipient|**SemCat: **landform > river**_ participate to coastal sediment transport

In conclusion, the meaning of the verb *drain* in the coastal engineering domain integrates at least two aspects, namely, the movement of matter and the change of possession of matter. Since both aspects are closely interrelated, *drain* is difficult to categorize since it can belong to the lexical domain of either movement or possession. However, it is true that specialized contexts foreground one of the aspects and relegate the other in the background. This type of fine-grained distinctions evidently required more careful analysis.

## Results

On the one hand, the percentages of annotated sentences classified into the predicate lexical domains were the following: The lexical domains of movement (24.67%), possession (20.78%), and change (16.89%) covered 62.34% of sentences. Next in the ranking were the lexical domains of existence (14.28%), action (9.10%), position (5.19%), manipulation (5.19%), and cognition (3.90%). The lexical domain of impact did not appear in our sample of sentences.

Coastal engineering is a process-oriented domain because it empirically describes and studies dynamic physical states (Faber et al., 2006). Therefore, dynamism is a phenomenon that pervades this domain, in which the interaction between different concepts is characterized by movement and change (León-Araúz, 2009, p. 24). For that reason, in our sample of annotated sentences, movement predicates (24.67%) and change predicates (16.89%) are among the most frequent. These predicates describe the natural and artificial processes of agents in named rivers, bays, and beaches, and the consequences of phenomena such as erosion or sedimentation. Furthermore, named rivers participate in a change of possession when they receive matters from plains and valleys, and then provide them to other entities, such as beaches and dunes, as they discharge into bays, seas, and oceans. Consequently, possession predicates (20.78%) are also predominant.

On the other hand, named rivers were also found to have a variety of semantic roles, namely agent, location, theme, patient, and recipient. Among the 1,716 arguments, filled with a named river and annotated with a semantic role, the most frequent one was agent (52.55%), and not location (23.08%) as expected. The theme and patient roles both occupied the third position in the ranking (10.26%). Recipient was the least frequent role for named rivers (3.85%), since there was less focus on the entity from which the rivers received matters (e.g., sediments, pollutants, or water). Instead, judging by the high percentage of possession predicates (20.78%), coastal engineering texts focused on the recipient entities that were provided with matter by the river (agent), primarily because they were directly affected by environmental problems such as erosion, pollution, or flooding, *inter alia*.

In the following, results are presented with regard to the semantic configuration of the eight lexical domains, and the semantic networks that arose from the semantic analysis.

### Lexical Domain of Movement

Four combinations of semantic roles were found for the lexical domain of movement (24.67% of sentences), shown in Table 4:

1. agent + patient: This pattern could express two relations, namely *drains* (i.e., a river flows along a place, while taking matter from it, and transports such matter to another place), or *moves_over* (i.e., a river flows over its banks, and thus inundates a town, a building, land, or crops). However, the semantic relation could always be identified, because of the semantic category of the concept that took the patient role. Accordingly, the pattern encoded the *drains* relation if the concept with the patient role was a landform (e.g., *watershed*), whereas the pattern conveyed the *moves_over* relation if the concept was part of water body (e.g., *riverbank*), spatial area (e.g., *town*, *land*, *crop*), or building (e.g., *temple*). Only the verb *drain* was found to transmit the *drains* relation, whereas several verbs could transmit the *moves_over* relation (e.g., *overflow*, *flow over*, *flood*, *inundate*, *drown*, and *submerge*).
2. agent + theme: This pattern always expressed the *transfers* relation, linking a named river to the matter it transports.
3. theme + location: This pattern conveyed either of two relations, namely *moves_into* (i.e., matter goes into a river, or more generally, into a landform), or *discharges_into* (i.e., a river meets the place of its mouth). Nonetheless, the semantic relation could always be differentiated, thanks to the semantic category of the concept with the theme role. As such, the pattern encoded the *moves_into* relation if the theme concept was matter (e.g., *bed sediment load*), whereas the pattern transmitted the *discharges_into* relation if the concept was a named river. Various verbs could express the *discharges_into* relation, namely *flow into*, *drain into*, *discharge into*, *debouche into*, *enter*, *reach*, and *meet*. The verb *drain*, followed by the preposition *into*, always referred to the place where a river mouth was located.
4. theme + path: This pattern always conveyed the *moves_across* relation, linking a named river to the landform across which the river flows.

**Table 4:** Results from the semantic annotations for the lexical domain of movement.

Verb lexical domain: movement						
Arg1	Arg2	Arg3	Example	Term	Relation	Term
**agent** Named River	**patient** entity> […] > landform		Natural sediment supply within this region is defined by the [**Ventura River**]_**agent**_, that *drains* large [**watersheds**]_**patient**_.	Ventura River	*drains*	watershed
Named River	entity > part > part of water body		The [**Salinas River**]_**agent**_ *overflows* its [**banks**]_**patient**_ and deposits sediments in the flood plain.	Salinas River	*moves_over*	bank
	entity > creation > structure > building		The [**temples**]_**patient**_, which date back to the thirteenth century b. c. e., were in danger of *being flooded* by the [**Nile River**]_**agent**_ during the construction of the Aswan High Dam.	Nile River		temple
	entity > space > area > administrative area		Rising 26 feet (10 m) above flood stage in some places, the [**Connecticut River**]_**agent**_ *submerged* at least four riverside [**towns**]_**patient**_, blighting their corn and grain fields, and caused deep financial hardship for their weary inhabitants.	Connecticut River		town
	entity > space > area > land		When the [**Nile River**]_**agent**_ *inundated* the [**land**]_**patient**_, the seepage naturally raised the water table.	Nile River		land
	entity > space > area > land		A crisscross network of earthen walls was formed in [**crops**]_**patient**_ that *would be flooded* by the [**Nile River**]_**agent**_.	Nile River		crop
**agent** Named River	**theme** entity > matter > solid matter		The low concentration values are indicative for the [**sediment load**]_**theme**_ *carried*/*transported* by the [**Weser River**]_**agent**_ before it enters the estuarine zone.	Weser River	*transfers*	sediment load
**theme** entity > matter > solid matter	**location** entity> […] > landform > natural water body		Thus, several tens of millions of [**bed load**]_**theme**_ *goes into* the [**North Passage of Yangtze estuary**]_**location**_ along with the ebb currents.	bed load	*moves_into*	Yangtze River Estuary
Named River	entity> […] > landform > natural water body		The [**Salinas River**]_**theme**_ *flows into*/*drains into*/*discharges into*/*debouches into*/*enters*/*reaches*/*meets* the [**Monterey Bay**]_**location**_.	Salinas River	*discharges_into*	Monterey Bay
**theme** Named River	**path** entity> […] > landform		The [**River Murray**]_**theme**_ *flows across* [**Tertiary formations**]_**path**_ to enter coastal lagoons behind the dune calcarenite barriers of Encounter Bay.	Murray River	*moves_across*	Tertiary formation

Depending on the ultimate application of the semantic annotations, the *moves_over*, *moves_into*, and *moves_across* relations could reasonably be collapsed into a single relation, namely the *has_path* relation.

### Lexical Domain of Possession

Table 5 summarizes the findings for the lexical domain of possession (20.78% of sentences). Two combinations of semantic roles were found:

1. agent + theme + recipient: This pattern expressed either of two relations, namely *gives/gives_to* (i.e., a named river supplies matter to a landform), or *receives/receives_from* (i.e., a named river takes matter from a landform while flowing along it). The specific semantic relation could always be specified, thanks to the semantic category of the concepts that took the agent and recipient roles. As such, if the agent was mentioned in the sentence and was a named river, the predicate conveyed the *gives/gives_to* relation. Nevertheless, if the recipient was mentioned in the sentence and was a named river, the semantic role pattern encoded the *receives/receives_from* relation.

**Table 5:** Results from the semantic annotations for the lexical domain of possession.

Verb lexical domain: possession						
Arg1	Arg2	Arg3	Example	Term	Relation	Term
**agent** Named River	**theme** entity > matter > **solid matter**	**recipient** entity > part of landform	The [**Salinas River**]_**agent**_ no longer *contributes* substantial [**beach size sand**]_**theme**_ to the [**Littoral Cell**]_**recipient**_.	Salinas River Salinas River	*gives* *gives_to*	beach size sand littoral cell
		entity > geographic feature > natural geographic feature > **landform** > natural water body	The [**Yellow Sea**]_**recipient**_ is influenced strongly by [**Yangtze River**]_**agent**_, which *discharges* more than 1.6 billion tonnes of [**sediments**]_**theme**_ annually.	Yangtze River Yangtze River	*gives* *gives_to*	sediment Yellow Sea
	entity > matter > **fluid matter** > water		The [**Changjiang River**]_**agent**_ *provides*/*supplies*/*brings*/*introduces*/*delivers* most of the [**fresh water**]_**theme**_ to the [**Changjiang Estuary**]_**recipient**_.	Changjiang River Changjiang River	*gives* *gives_to*	fresh water Changjiang Estuary
entity>[…] > landform	entity > matter > **fluid matter** > water	Named River	Normally, eutrophic conditions are caused by [**waters**]_**theme**_ *drained* by the [**Po River**]_**recipient**_ from the highly inhabited and cultivated [**Po plain**]_**agent**_.	Po River Po River	*receives* *receives_from*	water Po Plain
Ø	entity > matter > **solid matter**		Not all the [**sediments**]_**theme**_ *drained* by the [**Dee River**]_**recipient**_ participate to coastal sediment transport.	Dee River	*receives*	sediment
**recipient** entity	**theme** process > change > change in intensity > decrease	**location** Named River	However, in this instance, the [**anthropogenic effects**]_**recipient**_ probably dominate and *include* additional subsidence resulting from withdrawal of hydrocarbons, and the [**sediment supply reduction**]_**theme**_ in the [**Mississippi River**]_**location**_ by the construction of upstream impoundments and jetties that direct the riverine sediment offshore to deepwater.	sediment supply decrease	*takes_place_in*	Mississippi River

Whereas various verbs could transmit the *gives/gives_to* relation (e.g., *provide*, *supply*, *contribute*, *deliver*, *discharge*, *bring*, and *introduce*), only the transitive use of the verb *drain* expressed the *receives/receives_from* relation. In addition, the phrase that took the recipient role had to be frequently inferred from the whole sentence since this argument did not belong to the target predicate (see the example in the second row of Table 5, where the inferred recipient, *Yellow Sea*, is not an argument of the predicate *discharge*).

2. recipient + theme + location: This pattern always conveyed the *takes_place_in* relation, linking a process to its spatial and temporal dimensions. It must be clarified that the argument with the location role did not pertain to the same target predicate as the arguments with the recipient and theme roles. In other words, the argument with the theme role was filled with a deverbal noun designating a process, which acted as the predicate to which the argument with the location role belonged. A case in point is the sentence in the last row of Table 5, namely:

[Anthropogenic effects]_**recipient**_ **include** [sediment supply reduction]_**theme**_ in the [Mississippi River]_**location**_

The predicate *include* has *anthropogenic effects* and *sediment supply reduction* as its arguments. In turn, the nested predicate *reduction* has *Mississippi River* as an argument. The predicate *reduction* means *to change by decreasing*, therefore, it could have been classified as belonging to the lexical domain of change. However, since *reduction* is embedded in an argument structure that is steered by the predicate *include*, the decision was made to ascribe this type of sentence to the lexical domain of the higher-level predicate.

### Lexical Domain of Change

Table 6 summarizes the findings for the lexical domain of change (16.89% of sentences). Three combinations of semantic roles were found:

1. agent + patient: This pattern expressed one of three relations, namely, *improves* (i.e., an entity or a process changes an attribute or any other entity for the better), *worsens* (i.e., a process changes a river for the worse), or *affects* (i.e., an entity or a process causes a change in any other entity or process without producing a final result). However, the semantic relation was always evident, because of the semantic category of the concepts with the agent and patient roles. Accordingly, the pattern encoded the *affects* relation if the concept with the patient role was a landform (e.g., *Quanzhou Bay*), whereas the pattern conveyed the *worsens* relation if the concept with the agent role was a process increasing in size (e.g., *sea level rise*). In any other case, the pattern expressed the *improves* relation. The verbs *improve*, *enhance*, and *change* encoded the *improves* relation, whereas the verbs *affect*, *influence*, and also *change* encoded both the *worsens* and *affects* relations.
2. patient + location: This pattern could convey either of two relations, namely, *located_at* (i.e., an entity is located in a river), or *attribute_of* (i.e., a property that characterizes a river). Nevertheless, the semantic relation could always be differentiated, thanks to the semantic category of the concept with the patient role. As such, the pattern transmitted the *located_at* relation if the concept with the patient role was matter (e.g., *soft mud*), while the pattern expressed the *attribute_of* relation if the concept was an attribute (e.g., *discharge rate*).
3. patient + result: This pattern always encoded the *becomes* relation, linking a named delta or estuary that is transformed into any other landform.

**Table 6:** Results from the semantic annotations for the lexical domain of change.

Verb lexical domain: change						
Arg1	Arg2	Arg3	Example	Term	Relation	Term
**agent** entity > human > institution	**patient** Named River		The [**United States Army Corps of Engineers**]_**agent**_ adopted a modified version of his jetty plan for *improving* the [**St. Johns River entrance**]_**patient**_.	US ACE	*improves*	Saint Johns River Mouth
Named River	attribute		[**Salinas River Estuary**]_**agent**_ *helps improve* the [**quality of water**]_**patient**_ since it acts both as a filter […] and as a buffer between ocean storms and cities, protecting the inland areas from damage.	Salinas River Estuary	*improves*	water quality
process > change > transformation > restoration	Named River		[**Vegetation removal effect**]_**agent**_ over the entire study reach *changed* the [**Gila River**]_**patient**_ from a continually losing river for most years before clearing to a gaining stream during some months for most years following clearing.	vegetation removal effect	*improves*	Gila River
process > change > change in size > increase	Named River		[**Sea level rise**]_**agent**_ *changes*/*affects* [**Salinas River Estuary dynamics**]_**patient**_ and could thus potentially alter sediment supplies and process patterns.	sea level rise	*worsens*	Salinas River Estuary
**Explanation:** The above example allowed us to consider that the *sea level rise* process plays the agent role because it causes a change for the worse in the *Salinas River Estuary*. This process is really the result of other factors not mentioned in the example, such as the warming of the water and its subsequent expansion, the melting of glaciers, the sinking or rising of the land, the land movement, the pumping of freshwater from coastal aquifers resulting in sinking of the land, and the consolidation of the land (Dean and Dalrymple, 2004, p. 36–37). However, the *sea level rise* process was not annotated as taking the result role because the sentence does not contain any clues which lead to deem the process as the result of other processes.						
Named River	entity> […] > landform		The top of the [**Quanzhou Bay**]_**agent**_ is obviously *affected*/*influenced* by the [**Jinjiang River**]_**patient**_.	Jinjiang River	*affects*	Quanzhou Bay
**patient** entity > matter	**location** Named River		[**Soft muds**]_**patient**_ *are consolidating* at the [**mouth of the Mississippi River**]_**location**_.	soft mud	*located_at*	Mississippi River Mouth
attribute	Named River	**manner**	The [**discharge rate**]_**patient**_ at the [**Weser River**]_**location**_ *varies* [**greatly over a year**]_**manner**_, but a typical rate is in the vicinity of 200 m3/s.	discharge rate	*attribute_of*	Weser River
**patient** Named River	**result** entity> […] > landform		The [**Chandeleurs Islands**]_**result**_ *are remnants of* the [**Saint Bernard River delta**]_**patient**_, formed by the Mississippi River.	Saint Bernard River Delta	*becomes*	Chandeleurs Islands

### Lexical Domain of Existence

Five different combinations of semantic roles were found for the lexical domain of existence (14.28% of sentences), shown in Table 7:

1. agent + patient: This pattern could convey one of three relations, namely, *result_of* (i.e., a process or an entity is derived from other process), *worsens*, or *creates* (i.e., an entity causes another entity to exist). Nonetheless, the semantic relation could always be distinguished, thanks to the semantic category of the concept with the agent role. Accordingly, the pattern transmitted the *result_of* relation if the concept was a movement process (e.g., *sediment supply*). The *worsens* relation was conveyed if the concept was an addition process (e.g., *salinity intrusion*), and the *creates* relation was conveyed if the concept was a named river. The verbs *form* and *build* expressed the *creates* relation, whereas *result in* and *be/represent*/*constitute/become a problem/issue/challenge/trouble/matter* expressed the *worsens* relation.
2. agent + patient + result: This pattern always encoded the *worsens* relation.
3. agent + result + location: This pattern transmitted the *takes_place_in* relation.
4. agent + theme: This pattern conveyed the *causes* relation. In the corresponding row of Table 7, the verb *provide* in the example sentence has the sense *to cause something to happen, making it possible*, similar to *allow*, or *permit* (Faber and Mairal, 1999, p. 279). In other words, the sentence foregrounds that the Changjiang River causes the entry of fresh water into the region (accordingly, in this case, *provide* is ascribed to the lexical domain of existence). In contrast, the fact that the river supplies water recedes into the background (and so, in this context, *provide* is not regarded as possession verb).
5. theme + description + location: This pattern expressed one of two relations, namely, *attribute_of,* or *takes_place_in*. Nevertheless, the semantic relation could always be discriminated, thanks to the semantic category of the concept that took the theme role. Accordingly, the pattern encoded the *attribute_of* relation if the concept was an entity (e.g., a mathematical model or a named river), whereas the pattern transmitted the *takes_place_in* relation if the concept was a process (e.g., *sediment load variation*). In the corresponding rows of Table 7, the verb *show*, in both example sentences, has the sense *to cause something to exist in the perception of others* (Faber and Mairal, 1999, p. 279). For that reason, *show* fits into the lexical domain of existence, and not of perception.

**Table 7:** Results from the semantic annotations for the lexical domain of existence.

Verb lexical domain: existence						
Arg1	Arg2	Arg3	Example	Term	Relation	Term
**agent** process > movement > transport	**patient** Named River		The [**Tenryu River Delta**]_**patient**_ *has developed* owing to the abundant [**sediment supply**]_**agent**_ from the Tenryu R.	Tenryu River Delta	*result_of*	sediment supply
process > adittion	Named River	**condition** process > activity	For instance, [**salinity intrusion**]_**agent**_ *constitutes a problem* in the [**Yangtze Estuary**]_**patient**_ because of the [**vast area using water from the Yangtze**]_**condition**_.	salinity intrusion	*worsens*	Yangzte River Estuary
Named River	entity> […] > landform		The Chandeleurs Islands are remnants of the [**Saint Bernard River delta**]_**patient**_, *formed* by the [**Mississippi River**]_**agent**_.	Mississippi River	*creates*	Saint Bernard River Delta
**agent** process > movement > transport	**patient** Named River	**result** process > change > transformation > pollution	Thus, [**several tens of millions of bed load goes into the North Passage of Yangtze estuary along with the ebb currents**]_**agent**_, and [**it**]_**agent**_ *results in* a continuous [**siltation**]_**result**_ in [**North Passage of Yangtze estuary**]_**patient**_.	siltation	*worsens*	Yangtze River Estuary
**agent** process > change > change in intensity > decrease process > change > change in size > decrease	**result** process > change > change in size > decrease process > movement > soil movement	**location** Named River	After the dam construction, the [**sediment supply decrease**]_**agent**_ in the [**Tenryu River**]_**location**_ *resulted in* a [**delta coastline recession**]_**result**_. The [**consolidation of the land**]_**agent**_ at the [**mouth of the Mississippi River**]_**location**_ *is causing* the [**sinking of the land**]_**result**_ with respect to an absolute datum.	sediment supply decrease consolidation of the land	*takes_place_in*	Tenryu River Mississippi River Mouth
**agent** Named River	**theme** process > addition		The [**Changjiang River**]_**agent**_ *provides* most of the [**fresh water input**]_**theme**_.	Changjiang River	*causes*	fresh water input
**theme** Named River	**description** attribute > physical attribute > state	**time** Named Dam & process > activity	[**Before construction of the High Aswan High Dam**]_**time**_, the [**Nile Delta shore**]_**theme**_ *showed* [**fluctuating equilibrium**]_**description**_ between sediment supplied by the Nile River and the transport along the coast.	fluctuating equilibrium	*attribute_of*	Nile Delta
entity > information > representation > model	attribute > measurement	**location** Named River	Blackstone River draining into Narragansett Bay has been extensively dammed, and although not well quantified, [**models**]_**theme**_ *show* [**decreasing sediment load**]_**description**_ in the [**Blackstone River**]_**location**_.	decreasing sediment load	*attribute_of*	Blackstone River
process > change > change in intensity > decrease		Named River	The dramatical [**sediment load variation**]_**theme**_ in the [**Pearl River**]_**location**_, with the almost unchanged water discharge level, *represents* an [**example of such effect that human activities can have on river deltas**]_**description**_.	sediment load variation	*takes_place_in*	Pearl River

### Lexical Domain of Action

Three combinations of semantic roles were found for the lexical domain of action (9.10% of sentences), shown in Table 8:

1. patient + location: This pattern conveyed the *located_at* relation.
2. agent + description: This pattern transmitted the *has_function* relation, namely, a process or an entity (whether natural or artificial) that is linked to its specific function. In this case, a named river, delta, or estuary, despite being a natural entity, which is not goal-directed, is used for human profit (e.g., Salinas River Estuary *has_function* filter). Although this relation could also have been regarded as functional hyponymy (e.g., Salinas River Estuary *type_of (function)* filter), the annotators agreed to assign the *has_function* relation to this semantic role combination within the lexical domain of action.
3. patient + purpose: This pattern also expressed the *has_function* relation (e.g., Camboriú River *has_function* water supply). Like most sentences in which a named river is mentioned, the corresponding sentence in Table 8 with this combination of semantic roles is a high-density knowledge-rich context, namely, a context containing several terms of interest in a particular knowledge domain that are linked to other terms through different semantic relations (Meyer, 2001; León-Araúz and Reimerink, 2019). For instance, the sentence also conveys the following conceptual propositions associated with river damming, which reflect its multidimensionality. More specifically, although river damming is a beneficial activity because it assures the population of a water supply, it can also be regarded as a damaging activity because of its potentially adverse impact on the environment: damming *causes* coastal erosion; damming *worsens* beach; and damming *has_function* water supply.

**Table 8:** Results from the semantic annotations for the lexical domain of action.

Verb lexical domain: action						
Arg1	Arg2	Arg3	Example	Term	Relation	Term
**patient** entity > creation > structure > defence structure	**location** Named River		At least 90 dams over 60 m *are under construction*, including the [**Three Gorges dam**]_**patient**_ on the [**Yangtze**]_**location**_ (175 m).	Three Gorges Dam	*located_at*	Yangtze River
**agent** Named River	**description** attribute > ability		[**Salinas River Estuary**]_**agent**_ helps improve the quality of water since **it** *acts* both *as* a [**filter**]_**description**_ that catches nutrients, sediments, and even pollution, preventing them from moving further into the environment, and *as* a [**buffer**]_**description**_ between ocean storms and cities, protecting the inland areas from damage.	Salinas River Estuary Salinas River Estuary	*has_function* or *type_of (function)* *has_function* or *type_of (function)*	filter buffer
**patient** Named River	**purpose** process > addition		In the year 1950, the [**Camboriú River**]_**patient**_ *was dammed* for [**water supply**]_**purpose**_, reducing the sediment input to the beach, thus the **river damming** caused coastal erosion.	Camboriú River damming damming damming	*has_function* *causes* *worsens* *has_function*	water supply coastal erosion beach water supply

### Lexical Domain of Position

Table 9 summarizes the findings for the lexical domain of position (5.19% of sentences). Two combinations of semantic roles were found:

1. agent + theme + location: This pattern expressed the *places* relation, linking a named river to the matter that it deposits at a particular location. The argument with the theme role was always matter, whether solid (e.g., *sediment*) or fluid (e.g., *soft mud*). The verbs that frequently encoded this relation were *deposit* and *accumulate*.
2. theme + location: This pattern conveyed the *located_at* relation. In contrast to the pattern above, the argument with the theme role was either a landform (e.g., *salt marsh*) or a defense structure (e.g., *jetty*).

**Table 9:** Results from the semantic annotations for the lexical domain of position.

Verb lexical domain: position						
Arg1	Arg2	Arg3	Example	Term	Relation	Term
**agent** Named River	**theme** entity > matter	**location** entity>[…] > landform	The [**Salinas River**]_**agent**_ *deposits*/*accumulates* [**sediments**]_**theme**_ in the [**flood plain**]_**location**_.	Salinas River	*places*	sediment
**theme** entity> […] > landform	**location** Named River		The field site for this study is the [**Zuidgors salt marsh**]_**theme**_, *located in* the [**Western Scheldt estuary**]_**location**_ in The Netherlands.	Zuidgors salt marsh	*located_at*	Scheldt River Estuary
entity > creation > structure > defence structure			However, the construction of the [**jetty**]_**theme**_, *located on* the [**Camboriú River mouth**]_**location**_, changed the beach planform classification to natural beach reshaping or self-reshaping.	jetty		Camboriú River Mouth

### Lexical Domain of Manipulation

Table 10 summarizes the findings for the lexical domain of manipulation (5.19% of sentences). Only one combination of semantic roles was found, namely agent + patient. This pattern transmitted the *controls* relation, linking a named river to the process (e.g., *natural sediment supply*) or attribute (e.g., *shoreline characteristic*) that the river manipulates. The most frequent verb employed to express the relation was *define*.

**Table 10:** Results from the semantic annotations for the lexical domain of manipulation.

Verb lexical domain: manipulation						
Arg1	Arg2	Arg3	Example	Term	Relation	Term
**agent** Named River	**patient** process > movement > transport		[**Natural sediment supply**]_**patient**_ within this region *is defined* by the [**Ventura River**]_**agent**_ that drains large watersheds.	Ventura River	*controls*	natural sediment supply
	attribute		[**Shoreline characteristics**]_**patient**_ *are defined* by [**Santa Clara River**]_**agent**_.	Santa Clara River		shoreline characteristic

### Lexical Domain of Cognition

Table 11 summarizes the findings for the lexical domain of cognition (3.90% of sentences). Two combinations of semantic roles were found:

1. instrument + theme + location: This pattern encoded the *applied_to* relation, linking a mathematical model to the named river, whose magnitude was estimated by the model. The argument with the instrument role was always a mathematical model (e.g., *NOAH model*), and that of the theme role was a magnitude (e.g., *evaporation*).
2. theme + location + rate: This pattern conveyed the *attribute_of* relation. As in the pattern above, the argument with the theme role was a magnitude, but its value was specified by adding the rate role.

**Table 11:** Results from the semantic annotations for the lexical domain of cognition.

Verb lexical domain: cognition						
Arg1	Arg2	Arg3	Example	Term	Relation	Term
**instrument** entity > information > representation > model	**theme** attribute > measurement > magnitude	**location** Named River	Feng and Houser (2008) also find that the [**NOAH model**]_**instrument**_ *underestimates* [**evaporation**]_**theme**_ in the [**Mississippi River basin**]_**location**_.	NOAH model	*applied_to*	Mississippi River Basin
**theme** attribute > measurement > magnitude > level > mean	**location** Named River	**rate**	The [**average discharge rate of beach size sand**]_**theme**_ in the [**Salinas River**]_**location**_ *is estimated* at [**approximately 65,000 cubic yards per year**]_**rate**_.	average discharge rate of beach size sand	*attribute_of*	Salinas River

### From the Semantic Annotation to Semantic Frames

The semantic analysis of the predicate-argument structure of the sentences offered a comprehensive set of conceptual propositions. These propositions reflected the entities and processes that participated in the events educed by named rivers through predicates, and their interaction.

Furthermore, the sentences that preceded and followed our set of sentences were also semantically analyzed. This permitted us to construct the frames elicited by the rivers with a much broader explanatory adequacy. The semantic networks were then validated by a coastal engineering expert from the University of Granada (Spain).

In the following, the frame evoked by the Salinas River is depicted, and also the frame educed by the Dee, Mersey, Ribble, and Solway Firth estuaries.

#### Frame Evoked by the Salinas River

**Figure 3:**
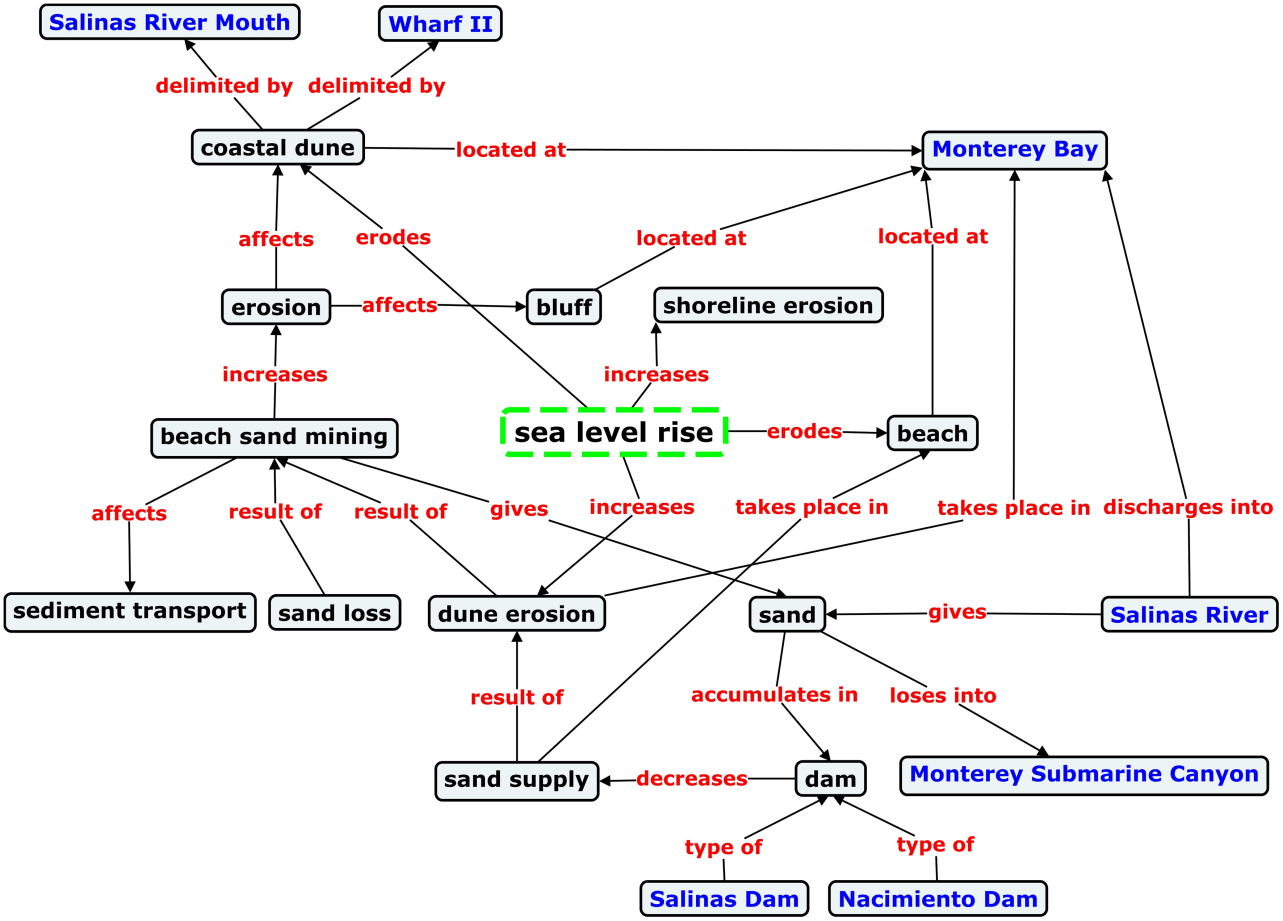
Semantic network evoked by the Salinas River, which also serves as the geographic contextualization of the sea level rise concept in the Coastal Engineering domain.

The Salinas River (California, the United States) evoked the frame shown in Figure 3. In this semantic network, Monterey Bay and Salinas River are two named entities, from different categories, associated with the same environmental problem depicted in the frame. Therefore, the semantic network fulfills Tobler’s (1970, p. 236) First Law of Geography, which states that “everything is related to everything else, but near things are more related than distant things.” In other words, since the Salinas River discharges into the Monterey Bay, both entities are spatially close and thus associated with the negative effects of sea level rise on shoreline erosion.

For the thematic description of the Salinas River, a textual explanation of its relational behavior was crafted from the semantic network as follows.

**Thematic description of the Salinas River**: Sediment is a resource essential both to the economic and environmental vitality of *Monterey Bay* beaches and to the mitigation of *shoreline erosion*. The sources of *sand* to the southern *Monterey Bay* are from the discharge of the *Salinas River* and from the erosion of the *beaches* and *coastal dunes*. However, human activities and natural processes are changing sand availability. Namely, *dams* constructed along the *Salinas River* have decreased its *sand supply*. Hence, most sediment from the river is driven north and potentially lost into the *Monterey Submarine Canyon*, and *beach sand mining* and *sea level rise* cause *dune erosion* to progress at a higher rate.

On the other hand, conceptual representations in TKBs can be enhanced when specialized concepts are embedded in situations (Meyer et al., 1992; Faber, 2011), for instance, situations geographically contextualized. As such, the representation of named rivers from coastal engineering in EcoLexicon enables the geographic contextualization of specialized concepts from that subject field in semantic networks. In this work, geographic contextualization consists in viewing a specialized concept from a situation in which the concept is related to specific named geographic entities, such as rivers and bays, because it is involved in an environmental problem which affects those geographic entities. Therefore, for the geographic contextualization of the sea level rise concept in the coastal engineering domain, EcoLexicon would show the semantic network in Figure 3. The situational elements in such a frame (i.e., concepts and semantic relations) would facilitate to represent and understand that sea level rise is causing dune erosion of Monterey Bay beaches to progress at such a high rate that the sediments discharged by the Salinas River are not enough to alleviate the coastal erosion of the bay. The frame in Figure 3 would also be valid for the geographic contextualization of any of the specialized concepts that are integrated into that network.

#### Frame Evoked by the Dee, Mersey, Ribble, and Solway Firth Estuaries

**Figure 4:**
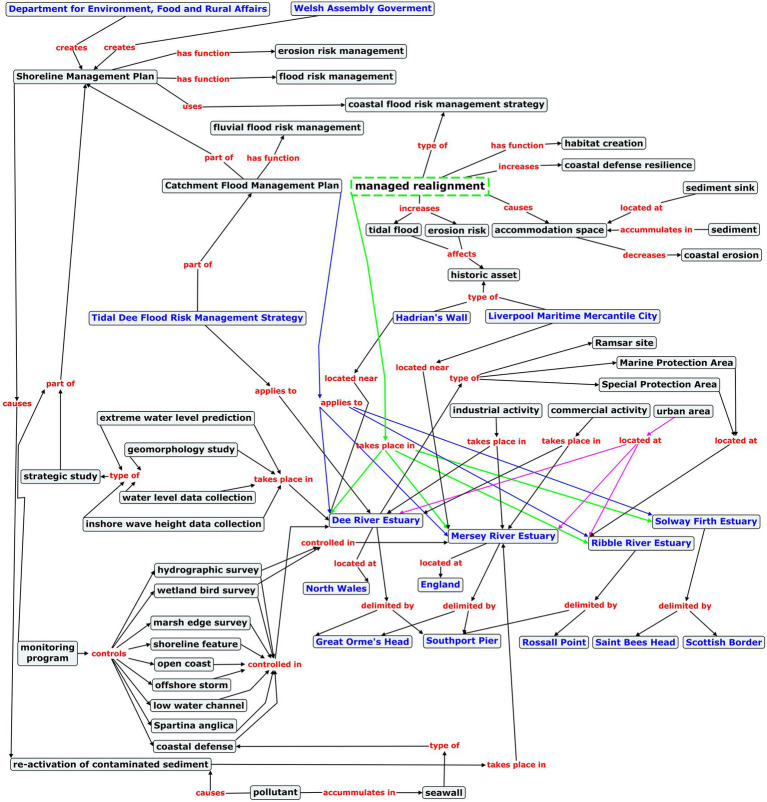
Semantic network evoked by the Dee, Mersey, Ribble, and Solway Firth estuaries, which also serves as the geographic contextualization of the managed realignment concept in the Coastal Engineering domain.

The Dee, Mersey, Ribble, and Solway Firth estuaries (in the United Kingdom) educed the frame in Figure 4. The four estuaries are spatially close and associated with the same environmental problem, according to Tobler’s (1970) First Law of Geography.

The thematic description of the estuaries was elaborated from the semantic network as follows.

**Thematic description of the Dee, Mersey, Ribble, and Solway Firth estuaries**: In Great Britain, the *Department for Environment, Food and Rural Affairs*, and the *Welsh Assembly Government* have required to produce *shoreline management plans* (SMPs) for the length of coastline which stretches from *Great Orme’s Head* in Wales to the *Scottish Border* on the *Solway Firth Estuary*, including the *Dee*, *Mersey*, and *Ribble* estuaries.

The overall aim of SMP is the *flood* and *erosion risk management* along the coast. Hence, SMP sets out policies for managing the coastline to reduce those risks to *urban areas*, *industrial* and *commercial activities*, and natural environments such as *marine protection areas*. One of those policies is the *managed realignment*, namely, removing *coastal defenses* or building new ones further inland to allow an area to become flooded by the sea. *Managed realignment*, usually pursued in estuarine areas, permits: The restoration of *accommodation space* containing *sediment sinks* for sediments mobilized by erosion; *habitat creation*, such as salt marshes and mud flats; and long-term *coastal defense resilience*. However, in areas where there are benefits in reverting to natural processes through *managed realignment*, there may be an increase in *tidal flooding* or *erosion risk* with associated negative impacts on *historic assets*.

Other plans, incorporated into the SMP, have been developed to coordinate works for flood and erosion risk management, such as *catchment flood management plans*, which predominantly consider *fluvial flood risks*. SMP also includes a *monitoring program* to check *shoreline features* and *wetland bird surveys*, among others, and *strategic studies*, for instance, for the *extreme water level prediction* in the *Dee River Estuary*.

For the geographic contextualization of a specialized concept such as managed realignment, EcoLexicon would show the semantic network in Figure 4.

## Discussion

It seems appropriate to embark on a discussion of the semantic behavior of named rivers in the coastal engineering domain. They are not solely conceptualized as the backdrop and scenario where human activities and environmental processes occur, as evidenced by the diversity of semantic roles that named rivers could play. The corpus data revealed that named rivers are generally conceived as *agents*. As such, they initiate natural processes, which in turn have an effect on or produce a result in another entity. Not surprisingly, coastal engineering texts attach major significance to the study of the processes that each named river triggers. For example, rivers deposit sediments, function as a filter to ameliorate pollution, and control shoreline characteristics. They are also deeply involved in the prevention of coastal erosion by supplying sand, and there exists a close relationship between rivers and bays in sediment concentration and transport.

Obviously, for the supply of sand, it is necessary for rivers first to act as *recipients* of sediments and water when draining valleys and plains.

As *patients*, named rivers undergo a change of condition for the better, when defense structures such as jetties, or processes such as vegetation removal, maximize the affordances offered by rivers. However, their conditions can also worsen when river damming and sea level rise cause the supply of fluvial sediment to decrease, or siltation pollutes rivers.

In the role of *theme*, rivers are subject to change. For instance, they can undergo a change of location, since, when flowing along their course, they cross other entities. They also participate in a change of possession, when they provide sediments to other entities, such as bays, beaches, and dunes, upon discharging into bays, seas, and oceans. Their nature may even change, when they become another type of landform (e.g., a river delta becomes an island). According to Faber and Mairal (1999), one of the most important environmental processes is change. In fact, the results showed that, in the context of named rivers, the change in sediment possession is predominant.

When rivers function as *locations*, the corpus examples specify the following: (1) the entities located on them, whether natural (e.g., *salt march*, *soft mud*) or artificial (e.g., *jetty*, *dam*); (2) the properties of the rivers (e.g., *discharge rate*, *evaporation*, *sediment load*, *runoff*); and (3) the mathematical models applied to predict the values of those properties (e.g., *NOAH model* for evaporation, *Grid-to-Grid model* for river runoff).

Consequently, from this discussion, one can infer that named rivers, at least in situations of specialized communication, are used in ways otherwise than to perform acts of reference. These findings point toward Evans and Wimmer’s (1990, p. 274), Searle’s (1969, and 1983, Ch. 9), Sjöblom’s (2006), and Strawson’s (1974/2004) claim as to context cannot be omitted in linguistic accounts of proper names, nor in terminological accounts of named entities relevant to a subject field.

## Conclusion

A set of 1,694 sentences, in which a potamonym was an argument of the predicate of the sentences, from a coastal engineering corpus were semantically analyzed and annotated with the lexical domain of the predicates, the semantic role and category of the arguments, and the semantic relation between the arguments. The aim was to propose a linguistic and terminological approach to the study of named entities in scientific discourse to represent them in a TKB within the framework of Frame-based Terminology, more specifically in EcoLexicon.

The semantic analysis and annotation of argument structures were powerful tools that effectively extracted usage information regarding named rivers in coastal engineering texts. The combination of lexical domains, semantic roles, categories, and relations generated frames that reflected the entities and processes that participated in the events educed by named rivers, and how they all interacted. Knowledge acquisition about named rivers could be conceived as a progressive expansion of meaning, which began at the phrase level, and resulted in the codification of entire semantic frames for named rivers to be represented in TKBs since those frames underlay the usage of named rivers in the corpus.

These propositional representations, derived from the analysis of predicate-argument structure, are a type of *tertium comparationis* that can be used as the basis for semantic equivalence in machine-translation applications (Buendía-Castro and Faber, 2016). In fact, the analysis evidenced that the predicates in the same lexical domain tended to combine with terms in the same or similar semantic categories such as matter, landform, process of restoration, process of change in increase, and magnitude attribute.

For the conceptualization of the behavior of named rivers in the coastal engineering domain, the representation of a large number of non-hierarchical relations was essential (e.g., *drains*, *discharges_into*, *gives*, *has_path*, *becomes*, *worsens*, and *creates*). These domain-specific relations make knowledge representation more meaningful and connected to reality because they are both multidimensional and dynamic (Faber et al., 2009, p. 16; León-Araúz, 2009, p. 149, 176, and 184). Coastal engineering is a process-oriented domain that studies dynamic physical states (Faber et al., 2006). Therefore, dynamism is a phenomenon that pervades this domain, whose representation requires the use of non-hierarchical relations.

Semantic networks facilitated for EcoLexicon to geographically contextualize those specialized concepts integrated into the river-evoked frames. In other words, the analysis of the local contexts of potamonyms (i.e., the analysis of predicate-argument structure of sentences that mention named rivers) allowed the transition to global contexts (i.e., semantic frames that depicted environmental problems) that encompassed the conceptual networks reflected in the texts as background situations for specialized concepts. Therefore, since context, knowledge, and reasoning are closely intertwined (Brézillon, 2005), it will be examined how the river-evoked frames can be applied to enhance the geospatial modeling of rivers in geographic information systems, as envisaged by Feng et al. (2004), Garrido and Requena (2011), and Lindenschmidt and Carr (2018).

In future research, the statistical analysis of the annotations will be carried out applying machine-learning techniques, specifically decision tree and random forest, to construct a predictive model. It is expected that the results will reveal which rules permit the prediction of the *semantic relation* between two arguments in a sentence from the predictor variables *verb lexical domain*, *semantic role*, and *semantic category*. This is a framework that has not been explored in terminology, and could be beneficial to the implementation of automatic systems that perform semantic annotation, and construction of semantic networks and thematic description of named entities in specialized discourse.

Furthermore, this study focused on the usage of named rivers in a coastal engineering corpus in English language. Therefore, the analysis of cross-cultural differences in conceptualization of this landform, in the same subject field, in other languages such as Spanish, German, and modern Greek is also deferred for further investigation. It is also planned the semantic analysis of colponyms (i.e., named bays), litonyms (i.e., named beaches and coasts), helonyms (i.e., named wetlands), and named protected areas in the coastal engineering.

Finally, another question that will be investigated in future work is the possibility to conceptualize a subject field in EcoLexicon considering the named entities, relevant to that specialized domain, as starting points for knowledge extraction from corpora and for conceptual analysis. Two of the phases of the workflow in terminology work is term extraction and term selection (Chiocchetti et al., 2013). Both phases could be performed in a specialized corpus taking into consideration the terms associated with relevant named entities, in a similar way to the procedure followed in this study for named rivers in a coastal engineering corpus.

## Data Availability Statement

The datasets presented in this article are not readily available because they are subject to ongoing research. Requests to access the datasets should be directed to juanrojas@ugr.es.

## Author Contributions

The author confirms being the sole contributor of this work and has approved it for publication.

## Funding

This research was carried out as part of two projects: (1) PID2020-118369GB-I00, “Transversal Integration of Culture in a Terminological Knowledge Base on Environment” (TRANSCULTURE), funded by the Spanish Ministry of Science and Innovation; and (2) A-HUM-600-UGR20, “Culture as Transversal Module in a Terminological Knowledge Base on the Environment” (CULTURAMA), funded by the Andalusian Ministry of Economy, Knowledge, Business, and University. Funding was also provided by an FPU grant given by the Spanish Ministry of Education to JR-G.

## Conflict of Interest

The author declares that the research was conducted in the absence of any commercial or financial relationships that could be construed as a potential conflict of interest.

## Publisher’s Note

All claims expressed in this article are solely those of the authors and do not necessarily represent those of their affiliated organizations, or those of the publisher, the editors and the reviewers. Any product that may be evaluated in this article, or claim that may be made by its manufacturer, is not guaranteed or endorsed by the publisher.

## Footnotes

^1^ http://ecolexicon.ugr.es

^2^ http://olst.ling.umontreal.ca/cgi-bin/dicoenviro/search_enviro.cgi

^3^ https://www.eionet.europa.eu/gemet/en/themes/

^4^ http://www.fao.org/faoterm/en/

^5^ http://aims.fao.org/en/agrovoc

^6^ http://www.environmentontology.org/Browse-EnvO

^7^The English EcoLexicon Corpus is publicly available in Open Corpora in Sketch Engine: https://www.sketchengine.eu/ecolexicon-corpus/.

^8^ http://www.geonames.org

^9^In the case of river basins, the term variants *basin* (British English), *catchment* (Australian English), and *watershed* (American English) were also manually searched in the corpus to identify proper names of this landform. This terminological variation for river basins is pointed out by Renschler (2004).

^10^We focused on the fact that verbs, whether specialized or general language, are crucial to meaning because they are generally what relate concepts in specialized texts. This study thus did not address the phenomena of terminologization or determinologization of verbs, although we recognize that both aspects need to be further investigated.

^11^The designation of the semantic roles for predicates could be different depending on the level of generality adopted (Van Valin, 2004). Another alternative would be possessor + theme + beneficiary.
